# Transforming Medicine: Cutting-Edge Applications of Nanoscale Materials in Drug Delivery

**DOI:** 10.1021/acsnano.4c09566

**Published:** 2025-01-17

**Authors:** Rumiana Tenchov, Kevin J. Hughes, Magesh Ganesan, Kavita A. Iyer, Krittika Ralhan, Leilani M. Lotti Diaz, Robert E. Bird, Julian M. Ivanov, Qiongqiong Angela Zhou

**Affiliations:** † CAS, a division of the American Chemical Society, Columbus, Ohio 43210, United States; ‡ ACS International India Pvt. Ltd., Pune 411044, India

**Keywords:** nanocarrier, drug delivery, nanoparticle, nanocrystal, nanoemulsion, nanotube, micelle, exosome

## Abstract

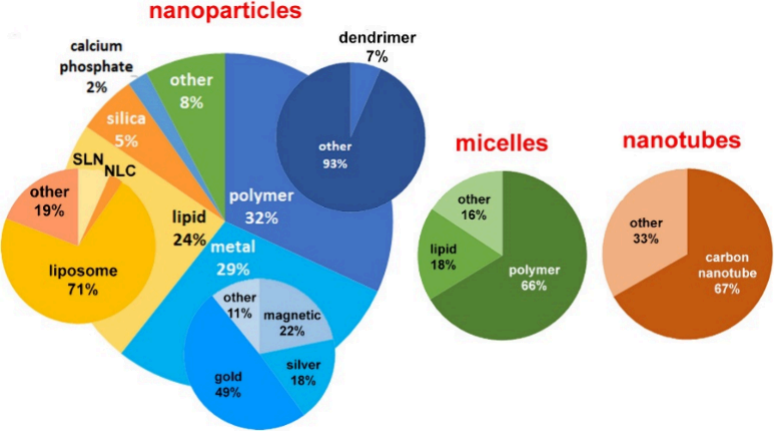

Since their inception in the early 1960s, the development and use of nanoscale materials have progressed tremendously, and their roles in diverse fields ranging from human health to energy and electronics are undeniable. The application of nanotechnology inventions has revolutionized many aspects of everyday life including various medical applications and specifically drug delivery systems, maximizing the therapeutic efficacy of the contained drugs by means of bioavailability enhancement or minimization of adverse effects. In this review, we utilize the CAS Content Collection, a vast repository of scientific information extracted from journal and patent publications, to analyze trends in nanoscience research relevant to drug delivery in an effort to provide a comprehensive and detailed picture of the use of nanotechnology in this field. We examine the publication landscape in the area to provide insights into current knowledge advances and developments. We review the major classes of nanosized drug delivery systems, their delivery routes, and targeted diseases. We outline the most discussed concepts and assess the advantages of various nanocarriers. The objective of this review is to provide a broad overview of the evolving landscape of current knowledge regarding nanosized drug delivery systems, to outline challenges, and to evaluate growth opportunities. The merit of the review stems from the extensive, wide-ranging coverage of the most up-to-date scientific information, allowing unmatched breadth of landscape analysis and in-depth insights.

## Introduction

The development and use of nanotechnology have grown substantially in the past decades, gaining impressive momentum. The application of nanotechnology inventions or products has revolutionized many aspects of everyday life including various medical applications and specifically drug delivery systems (DDSs), maximizing the therapeutic efficacy of the contained drugs by means of bioavailability enhancement or minimization of adverse effects.

Overall, drug delivery has been a complex challenge, often impeded by the limited solubility, stability, and bioavailability of many therapeutic agents. These constraints have motivated a widespread quest to find more efficient ways to deliver drugs to their intended targets. Among the transformative advancements in drug delivery technologies, nanosized DDSs (nano-DDSs) have emerged as a formidable force in the world of pharmaceutical science and practice offering a dynamic range of solutions that transcend traditional pharmaceutical boundaries.

Typically, nanosized objects contain a small number of atoms or molecules, a significant part of which are located on their surface. Therefore, while the characteristics of macrosystems are determined by their bulk properties, for nanosystems, the surface effects are dominant. Unlike macrosystems, the properties of which generally do not depend on their size, the properties of the nanosized systems are essentially dependent on their size. As a result, nanoscale particles exhibit specific structural, chemical, mechanical, magnetic, electrical, and biological properties, which are often of significant value in their applications as DDSs. Prominent characteristics of the nanosized DDSs are their high surface area/volume ratio, their chemical and geometric tunability, and their ability to interact with biomolecules in order to facilitate uptake across the cell membrane. The large surface area promotes high affinity for drugs and small molecules, such as ligands or antibodies, to bind and adsorb, facilitating targeting and controlled release.

Due to the advantage of their size, nanoscale systems have been demonstrated to be efficient DDSs and may be useful for encapsulating drugs, enabling more precise targeting with a controlled release. Their use may address some of the most pressing challenges in drug delivery, such as solubilizing poorly water-soluble drugs, protecting labile drugs from degradation, and delivering drugs selectively to disease sites. Nanosized structures stay in blood circulation for a prolonged time, allowing the sustained release of incorporated drugs. Thus, they cause fewer plasma fluctuations with reduced adverse effects.^1^ Being nanosized, these structures penetrate tissue, facilitate easy uptake of the drug by cells, enable efficient drug delivery, and ensure activity at the targeted location. The uptake of nanostructures by cells is much higher than that of large particles.^2,3^ Hence, they directly interact to treat diseased cells with improved efficiency and reduced side effects. Modifying or functionalizing nanoparticles to deliver drugs through the blood–brain barrier for targeting brain tumors has been one superb outcome of medical nanotechnology.^4^ Furthermore, due to their size, shape, and functionality, nanoparticle systems are crucial components of DNA delivery vectors.^4,5^ They can penetrate deep into tissues and are absorbed by the cells efficiently.^6^ Moreover, nanoparticles have widened the scope of pharmacokinetics for insoluble drugs.

In this review, we utilize the CAS Content Collection, a vast repository of scientific information extracted from journal and patent publications, to analyze trends in nanoscience research relevant to DDSs in an effort to provide a comprehensive and detailed picture of the use of nanotechnology in this field. We examine the publication landscape in the area in an effort to provide insights into advances and developments in current knowledge. We review the major classes of nanosized DDSs, their delivery routes and targeted diseases. We outline the most discussed concepts and assess the advantages of the various nanocarriers. The objective of this review is to provide a broad overview of the evolving landscape of current knowledge regarding nanosized DDSs, to outline challenges, and to evaluate growth opportunities, in order to further efforts in solving the problems that remain. The merit of the review stems from the extensive, wide-ranging coverage of the most up-to-date scientific information, allowing unmatched breadth of landscape analysis and in-depth insights.

This review is part of a series of articles based on the CAS Content Collection aimed to identify emerging topics in the field of nanotechnology. This involves understanding trends, such as the growth of certain topics over time, as well as establishing relationships between emerging topics. We achieved this by using a host of strategies including a quantitative natural language processing (NLP) approach to identify multiple emerging topics and subtopics across three major categories, materials, applications, and properties, by surveying over 3 million publications in the nanoscience landscape. This wealth of information has been condensed into several conceptual mind maps and other graphs, which will be published separately, providing metrics related to the growth of identified emerging concepts, grouping them into hierarchical classes, and exploring the connections between them. Our extensive analysis taking advantage of a NLP-based approach along with robust CAS indexing provides valuable insights in the field that we hope can help to inform and drive future research efforts.

## Advantages of Nanosized Drug Delivery Systems

The rationale behind employing nano-DDSs lies in the numerous advantages they offer compared to traditional drug delivery methods, which contribute to improved therapeutic outcomes, reduced side effects, and enhanced patient compliance. The advantages as well as certain disadvantages of the nano-DDSs are summarized in Table 1.

**Table 1:** Summary of the Advantages and Disadvantages of Nano-DDSs

Advantages	Disadvantages
• Nanosized carriers can be engineered to target specific cells, tissues, or organs. This **targeted delivery** minimizes the exposure of healthy tissues to the drug, concentrating its effects at the intended site of action.^7,8^	• Toxicity and biocompatibility issues: Some nanomaterials, particularly metallic NPs (e.g., gold, silver), may accumulate in the body and cause long-term toxicity or adverse immune reactions.^27,28^
• Many drugs, especially those with poor water solubility, can have limited bioavailability. Nano-DDSs can improve drug solubility, leading to **enhanced bioavailability** and, consequently, improved therapeutic efficacy.^8,9^ Thus, nanotechnology may help with drug repurposing: drugs whose development might have been abandoned due to poor bioavailability, which otherwise show satisfactory activity against intended targets, can be repurposed with the help of nanotechnology.^10,11^	• Challenges in large-scale production: Scaling up NP production from laboratory to commercial levels with consistent quality and safety is challenging and costly.^29,30^
• Nanocarriers can extend the time a drug circulates in the bloodstream. This **prolonged circulation time** contributes to a sustained release of the drug, reducing the frequency of administration and improving patient compliance.^12,13^	• Short circulation time and premature clearance: NPs can be recognized as foreign objects by the immune system and removed from circulation by the reticuloendothelial system (RES) before reaching their target.^31−33^
• Nanocarriers can **protect drugs** from degradation, metabolism, or elimination before reaching the target site. This protection enhances the stability of drugs and ensures a higher concentration reaches the intended location.^12,14,15^	• Regulatory and approval hurdles: Regulatory approval for nanomedicines is complex due to the novel materials and mechanisms involved, requiring extensive safety, toxicity, and efficacy data.^34,35^
• By selectively delivering drugs to the target site, nano-DDSs can minimize exposure to healthy tissues, reducing the potential for **toxicity** and lessening the occurrence of **side effects** commonly associated with systemic drug administration.^7,8^	• Stability and storage issues: Many NPs, especially biological-based ones (e.g., liposomes, polymeric micelles), may have limited stability during storage, which can lead to aggregation or drug leakage.^36,37^
• Nano-DDSs allow for the simultaneous delivery of multiple drugs. This is particularly beneficial for **combination therapy**, where different drugs with complementary mechanisms of action can be delivered together for a synergistic therapeutic effect.^16,17^	• Complex interactions with biological systems: NPs can interact unpredictably with biological systems, including proteins in the bloodstream, forming a “protein corona” that can alter their behavior and targeting ability.^38,39^
• Nanosized carriers can **overcome biological barriers**, such as the blood–brain barrier, allowing drugs to reach and act on specific locations that are otherwise difficult to access.^18,19^	• High development costs: The research, development, and clinical testing of nano-DDSs are resource-intensive, requiring multidisciplinary teams and advanced technologies.^40,41^
• Nanocarriers provide **flexibility in loading** a variety of drugs, including small molecules, proteins, nucleic acids, and imaging agents. This versatility makes them suitable for a wide range of therapeutic applications.^12,20^	• Patient safety and long-term effects: There is limited data on the long-term effects of NPs in the body, particularly concerning their accumulation and potential toxicity after repeated use.^28,42^
• The size and surface properties of nanocarriers can be tailored to **optimize pharmacokinetics**, leading to improved drug distribution, absorption, and elimination.^9,15^	
• Nano-DDSs can be designed for **personalized medicine** approaches, where treatments are tailored to individual patients based on diagnostic information. This customization can lead to more effective and targeted therapies.^21,22^	
• Some nanosized carriers can be designed to **combine therapeutic and diagnostic functions (i.e., act as theranostics)**, allowing for simultaneous imaging and treatment. This integration can provide real-time information about treatment efficacy.^23,24^	
• The reduced side effects, less frequent dosing, and improved efficacy associated with nano-DDSs contribute to enhanced **patient compliance** with prescribed treatment regimens.^25,26^	

## Key Nano-DDS Forms, Materials, and Applications

**Figure 1:**
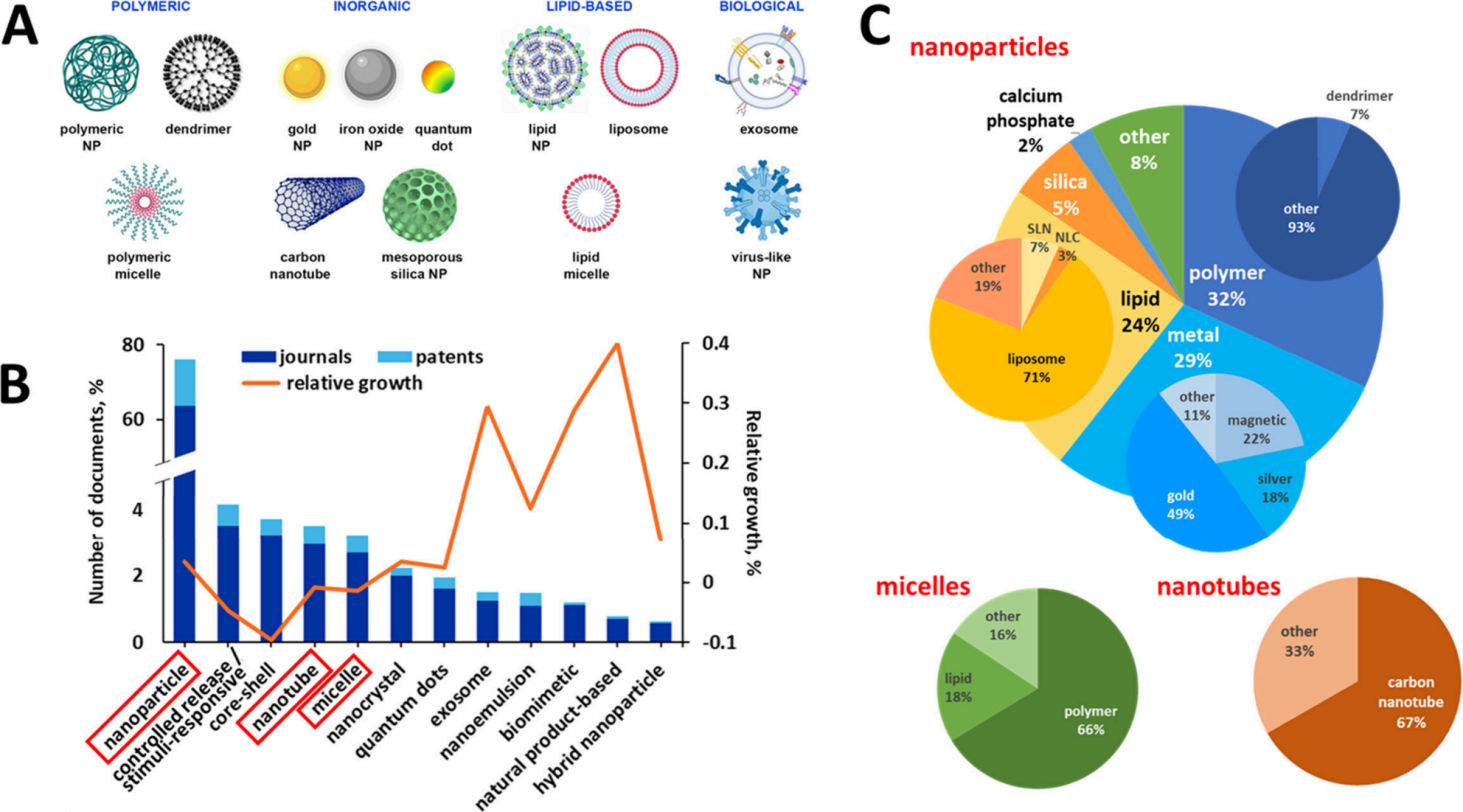
(A) Schematic representation of various types of nano-DDSs (some individual icons sourced from www.biorender.com). (B) Percentage of documents (journal articles and patents, blue bars) and relative growth (orange line; calculated as the increase in the number of documents in the last three years normalized over the total number of documents for the given nano-DDS type) related to various nano-DDS types; the red rectangles indicate the DDS represented on the right. (C) Distribution of documents related to NP (with the lipid, polymeric, and metal NP subcategory shares), micelle (with polymer and lipid shares), and nanotube (with carbon nanotube share) DDSs.

Since its dawn, nanotechnology has become a focus and a vital part of pharmaceutical science and has found numerous remarkable applications in drug delivery. For example, the potential of liposomes as DDSs was recognized almost immediately after their discovery in the 1960s.^43−47^ The use of the term “nanoparticle” in the context of drug delivery dates as far back as 1978.^48^ Continued interest resulting in extensive research and development has led to a wide variety of nano-DDS forms. Analysis of >600,000 publications allowed identification of the nano-DDS forms, a few representative examples of which are shown in Figure 1A, and their trends, in terms of both growth in publications (Figure 1B) and distribution of certain nano-DDS across subcategories (Figure 1C).

### Nanoparticles

Nanoparticles (NPs) are sub-micrometer-sized colloidal particles with distinctively tunable properties, selectively designed for specific applications. Pharmaceutical NPs constitute currently the best explored, mature area of nano-DDSs, with the highest number of published documents (Figure 1A). Nanoparticulate DDSs are intended to maximize drug efficacy and minimize cytotoxicity. A particularly important design feature of NPs for drug delivery is their surface functionalization accomplished by bioconjugation or passive adsorption of molecules onto the NP surface. Responsive biomaterials are powerful tools for controlling treatments such as cancer immunotherapies by providing precise control over the delivery and kinetics of therapeutic cargoes.^49^ Better efficacy and lower toxicity are often achieved by functionalizing NP surfaces with ligands that improve drug binding, suppress immune response, and afford targeted/controlled release.

The composition of the NP is chosen with respect to the target environment or anticipated effect. For example, biodegradable NPs can be designed to degrade upon delivery, reducing their bioaccumulation and toxicity.^50^ Metal NPs have optical properties that allow for less invasive imaging techniques.^50,51^ Plasmonic metal NPs are used as superior optically stable bioimaging agents for the early diagnosis of diseases. The photothermal response of NPs to optical stimulation can be exploited in tumor therapy.^52^

**Polymeric NPs** are currently the most popular class of NPs in drug delivery accounting for 32% of documents in the nano-DDS data set in the CAS Content Collection (Figure 1C). They are beneficial for drug delivery because they can be modulated with adequate physical properties, encapsulants, and surface ligands; they can also be tailored to co-deliver multiple therapeutic agents.^53^ Various stimuli-responsive (e.g., enzyme-, pH-, and redox-responsive) polymers, including natural and synthetic polymers, have been utilized as smart nanocarriers for drug delivery. Redox-responsive polymeric nanohydrogels exhibiting tissue-like mechanical properties and high porosity have been extensively studied and shown to be effective in protecting payloads, including protein drugs, gene therapeutics, and small-molecule drugs, in blood circulation as part of a strategy for controlled release.^54^ The most commonly used natural polymers, polysaccharides, include cellulose and its esters, chitosan, alginic acid/sodium alginate, hyaluronic acid, dextran, and xanthan gum, while poly(ethylene glycol) (PEG) and its copolymers such as poloxamines, poloxamers and also polystyrene, poly(ethylene terephthalate), poly(methyl methacrylate), poly(vinylpyrrolidone) (PVP), polyacrylamide, poly(vinyl alcohol), and polysorbates, as well as the biodegradable poly(lactic acid), polycaprolactone, and poly(lactic-*co*-glycolic acid), are preferred synthetic polymers.^53^

**Lipid NPs**(55) are widely explored and used nanocarriers (Figure 1C), contributing to 24% of publications in the NP DDS subset. Lipid-based NPs have been applied in drug delivery since the discovery of liposomes, which are spherical vesicles with lipid bilayers surrounding an aqueous core, in the 1960s. Subsequently they exhibited several significant advancements: (i) with the introduction of PEGylation,^56^ which increased their circulation half-lives further improving their efficiency;^57^ (ii) with the discovery of the cationic/ionizable liposomes able to deliver anionic nucleic acids;^55,58^ (iii) with the development of the solid lipid NPs (SLNs), consisting of solid lipids, and nanostructured lipid carriers (NLCs), combining solid and liquid lipids, offering enhanced drug-loading capacity and flexibility, higher stability, and largely improved scalability.^59,60^ A strong advantage of lipid NP drug carriers is the fact that most of their components are physiological lipids and excipients which are generally recognized as safe (GRAS).^61^ They are superior to other nano-DDSs in minimizing systemic toxicity while maintaining adequate solubility^62^ and constitute a common type of nanomedicines with regulatory approval.^63^ Lipid-based NPs can successfully deliver small molecules as well as protein and nucleic acid therapies *in vivo* to achieve remarkable activity. Their elegance lies in their ability to overcome some of the most pressing challenges in drug delivery: improving the solubility of poorly water-soluble drugs, protecting labile compounds from degradation, and precisely targeting disease sites within the body.^47,59,60,64−72^ Lipid NPs have a wide range of applications, including cancer therapy (reducing systemic toxicity and enabling targeted delivery), infectious disease treatment (improving drug stability and selective delivery to infected tissues), vaccine delivery (enhancing immune responses), targeting pulmonary endothelium for vascular repair after viral injury,^73^ precision treatment of viral pneumonia through macrophage targeting,^74^ mRNA delivery to the lungs carrying Cas9-based genetic editors,^75^ mRNA delivery to the bone microenvironment,^76^ and gene therapy (safe and efficient gene transfection).^55,77^ They are employed also in the treatment of neurological disorders (overcoming blood–brain barrier challenges), ophthalmic conditions (enhancing drug retention in the eye), cardiovascular therapies (improving drug solubility and controlled release), and more.

**Inorganic NPs** provide an appropriate framework in which multiple modules can be combined to give multifunctional capabilities. Inorganic materials such as metals (gold, silver, iron, and others), silica, calcium phosphate, and others (Figure 1A) have been used to prepare NPs for various drug delivery and imaging applications. Metallic NP formulations are particularly advantageous because of their potential for dense surface functionalization and for optical or thermal based therapeutic and diagnostic methods.^78^ Inorganic NPs have distinctive physical, electrical, magnetic, and optical properties. Particularly, plasmonic metal NPs (i.e., gold, silver, etc.) distinguish themselves from other inorganic NPs based on their distinctive optical property known as localized surface plasmon resonance^79,80^ originating from photon confinement to a nanosize particle, which property has been widely utilized for biological and medical applications.^81−83^ Gold NPs, for example, have been fabricated into various forms including nanospheres, nanorods, nanostars, nanoshells, and nanocages.^84,85^ Gold NPs have oscillating free electrons at their surface endowing them with photothermal properties.^80,86^ They are also easily functionalized, providing them with additional beneficial delivery capacities.^84^ Iron oxide NPs are another kind of metal NPs which make up the majority of US FDA-approved inorganic nanomedicines.^87^ Magnetic NPs comprising magnetite (Fe_3_O_4_) or maghemite (γ-Fe_2_O_3_) exhibit superparamagnetic properties at certain nanosizes and have been successfully used in imaging, drug delivery, and thermosensitive medications.^78,88^ Other commonly used inorganic NPs include mesoporous silica NPs, which have been successfully applied for gene and drug delivery.^89,90^

- **Magnetic iron oxide NPs**. Iron oxide NPs can generate heat when exposed to an alternating magnetic field, a property that has been utilized to induce cell death and stimulate an immune response in hyperthermia-based cancer treatment.^91^ Iron oxide NPs can also be used as contrast agents for magnetic resonance imaging (MRI), allowing for noninvasive tracking of immune cell migration and infiltration into tumor sites. In order to enhance their cellular uptake and effectiveness, these NPs can be modified with a specific coating, can be conjugated to drugs, proteins, enzymes, antibodies, or nucleotides, and can be directed to an organ, tissue, or tumor site using an external magnetic field. They can be also used in the development of dual-purpose probes for the *in vivo* transfection of siRNA.^92^
- **Silver NPs** known for their antibacterial activity, are also known to enhance the antitumor effects of anticancer drugs in combination therapies, allowing use of lower doses to reduce cytotoxic effects and increase efficacy.^93^ They can thus operate as direct anticancer agents, as well as delivery platforms of various cytotoxic drugs or enhance the anticancer performance of combinational partners upon chemo- or radiotherapy.^94^ Silver NPs can exhibit a plasmon resonance effect and generate heat when exposed to specific wavelengths of incident light.^95^ This property can be harnessed for photothermal therapy, where the localized heat generated by the NPs can selectively damage cancer cells and stimulate immune response. The plasmonic properties can be varied by changing the composition, size, and shape of the metallic NPs which can affect the collective oscillation of free electrons at their localized surface plasmon resonance wavelengths when irradiated with resonant light over most visible and NIR regions.^96,97^ Endowed with a tunable optical response, the NPs can be utilized as highly bright reporter molecules, effective thermal absorbers, and nanoscale antenna, via modulating the local electromagnetic field to detect changes in the environment.^98^
- **Gold NPs**. Possessing multifunctional therapeutic modalities, gold NPs can be used as targeted delivery systems for vaccines, nucleic acids, and immune antibodies, as theranostic agents, and in cancer therapy. They have also been successfully applied in medical imaging, such as radiotherapy, magnetic resonance angiography, and photoacoustic imaging. Gold nanostructures including NPs, nanorods, nanocages, etc., are easily synthesizable in diverse shapes and sizes through various chemical, physical, or biological methods, which empowers their manageability, since even minor modifications of their size and shape can produce significant alterations in their functional properties including biodistribution, metabolism, cytotoxicity, and immunogenicity.^99,100^ Similar to silver NPs, gold NPs can be utilized in photothermal therapy via localized surface plasmon resonance and are considered an excellent biomedical diagnostic tool.^99−102^ Recent progress in nanotechnology has afforded plasmonic gold NPs with tunable optical properties by manipulating parameters such as size, shape, and composition, which has attracted much interest for biomedical applications, especially for diagnostic imaging and drug delivery.^103−106^ A noteworthy application of gold and other inorganic NPs is in the so-called “spherical nucleic acids”:^107^ densely packed and highly oriented arrangements of linear nucleic acids forming a shell on an inorganic NP core (gold in the initial version^108^) in a three-dimensional, spherical geometry.^109^ Silver, iron oxide, silica, and semiconductor materials have also been used as inorganic cores in later versions of the spherical nucleic acids.^110−113^ Intracellular gene regulation, immunotherapy agents, and intracellular probes are among the suggested applications of these exotic nanostructures.^114−118^
- **Silica NPs**. Mesoporous silica exhibits high porosity, appropriate biocompatibility, and facile surface functionalization. Silica NPs can be engineered to various shapes, sizes, and surface properties, making them versatile tools for targeted drug delivery, imaging, and immunomodulation.^119^ After the introduction of a sub-micrometer mesoporous silica termed MCM-41^120^ and its successful application as a nanocarrier,^121^ it has been regarded as a promising DDS.^119^ Moreover, mesoporous silica exhibits a self-adjuvant property, significantly enhancing anticancer immunity without additional immunomodulators.^122^ Mesoporous silica has emerged as a prospective nanocarrier for cancer vaccines as well,^122^ imparting antitumor effect through dual loading of antigen and adjuvant on a single platform.^119^

### Nanocrystals

Certain drugs are highly insoluble, not only in aqueous solvents but also in lipids or oils due to their strong crystalline lattice energy. They are frequently formulated as nanocrystals, since the reduction in particle size through nanonization can overcome or improve solubility issues. Such nanocrystalline drug technology involves the reduction in the bulk size of the drug particles down to the nanosize range, thus altering their physicochemical properties, including enhancing drug bioavailability.^123^ Nanocrystals are carrier-free drug NPs surrounded by stabilizers such as polymers or surfactants and suspended in aqueous medium.^124^ Among the polymeric stabilizers, the most widely used are poloxamers (e.g., Pluronic F68, Pluronic F127), poly(vinyl alcohol), PVP, and cellulose derivatives (hydroxypropyl methylcellulose, hydroxypropyl cellulose). Among surfactants, Tween 80, sodium lauryl sulfate, and others, have been widely used.^125,126^ Due to high drug loading, nanocrystals exhibit effective therapeutic concentration to produce desirable pharmacological action. In addition to therapy, nanocrystal technology can be applied also in diagnostics.^127−129^ Examples of nanocrystalline drugs on the market include Rapamune (Wyeth), an mTOR inhibitor immunosuppressant especially useful in preventing transplant rejection; Emend (Merck), preventing nausea and vomiting caused by certain anticancer chemotherapy medicines; Tricor (Abbott) and Triglide (Sciele Pharma), both lowering cholesterol and triglyceride levels in blood; and Megace ES (Par Pharmaceutical), used to increase appetite and prevent weight loss in patients with AIDS.^123^

### Nanoemulsions

Emulsions are liquid–liquid dispersions with one liquid phase dispersed in the other liquid phase as small droplets with the droplets being nanosized in the case of nanoemulsions. Surfactants play a critical role in producing and stabilizing nanoemulsions by residing at the interface between the two immiscible phases.^130^ Nanoemulsions can be easily produced at a large scale using industrial methods including high-pressure homogenization and ultrasonication. Because of their small size and easily dispersible components with different hydrophobicity (e.g., hydrophobic drugs in the dispersed oil phase and hydrophilic proteins in the continuous aqueous phase), they are considered promising drug delivery vehicles to deliver hydrophobic drugs, and have been used as adjuvants for vaccines, demonstrating their clinical significance.^130−132^

### Nanotubes

Carbon nanotubes are successful drug and gene delivery platforms that can be functionalized with a variety of biomolecules, including antibodies, proteins, or nucleic acids, allowing for specific payload targeting particular tissues, organs, or cells. Carbon nanotubes are easily internalized by cells through passive and endocytosis-independent mechanisms, delivering drugs to the cytoplasm or nucleus. Nanotubes maintain a perpendicular position with respect to the cell membrane during uptake, perforating and diffusing through the lipid bilayer to move into the cytoplasm.^133^ Carbon nanotubes are large molecules, consisting of a repeating pattern of hexagonally arranged hybridized carbon atoms wrapped into a cylinder approximately 2.5–100 nm in diameter. Carbon nanotubes can be single-walled or multiwalled depending on the number of layered carbon sheets in their structure.^134^

Carbon nanotubes have been used as carriers of anticancer drugs, such as docetaxel, doxorubicin, methotrexate, paclitaxel, gemcitabine, anti-inflammatory drugs, osteogenic dexamethasone, steroids, and others. The distinctive optical properties of carbon nanotubes are the reason for their use in phototherapy.^135^ The effortless surface functionalization of carbon nanotubes has motivated their use in gene delivery as delivery vectors for plasmid DNA (pDNA), micro-RNA (miRNA), and small interfering RNA (siRNA). Despite great promise, carbon nanotubes possess a few disadvantages such as poor aqueous solubility and high cost as well as sustained and substantial concerns regarding their biodegradability with efforts being made to minimize these drawbacks.^136,137^

### Micelles

Micelles are colloidal systems formed by the self-assembly of amphiphilic molecules in aqueous media at concentrations above their critical micelle concentration. They comprise a hydrophobic core and a hydrophilic shell. The most widely used amphiphiles are lipids or polymers; thus the resultant micelles are either lipid micelles, polymeric micelles, or lipid–polymeric hybrid micelles. **Polymeric micelles** are made of amphiphilic block copolymers that self-assemble to form a core–shell structure in aqueous solution. The hydrophobic core can be loaded with hydrophobic drugs such as camptothecin, docetaxel, and paclitaxel, while the hydrophilic shell makes the whole system soluble in water and stabilizes the core.^8^ The most commonly used polymers for micelle formation are amphiphilic diblock copolymers such as polystyrene–PEG and triblock copolymers such as poloxamers, with graft and ionic copolymers (e.g., PEG–poly(ε-caprolactone)-*g*-polyethylenimine) used in some circumstances.^138−142^ The hydrophilic part is most often composed of PEG, but other polymers such as PVP, poly(acryloylmorpholine), or poly(trimethylene carbonate) have been also exploited; the hydrophobic segment can be made up of poly(propylene oxide), polyesters such as poly(ε-caprolactone), or homopolymers and copolymers of glycolic and lactic acids.

While liposomes have a lipid bilayer structure encapsulating an aqueous moiety, **lipid micelles** consist of a monolayer with the lipophilic chains forming the inner core and the hydrophilic heads exposed to the aqueous environment. The nanoscale dimensions and the hydrophilic shell protect them from elimination by the reticuloendothelial system, thereby increasing their circulation time and ability to deliver drugs to the targets.^143^**Hybrid micelles** prepared from lipid–polymer conjugates comprising water-soluble polymers, such as PEG or PVP, conjugated with phospholipids or long-chain fatty acids have been used to deliver various poorly soluble anticancer agents.^144^ For example, micelles formed by conjugates of phosphatidylethanolamine (PE) with PEG of various molecular weights, e.g., PEG750–PE, PEG2000–PE, and PEG5000–PE, have been reported to accumulate efficiently in tumors.^145^

### Natural Product-Based Nano-DDSs

The class of natural product-based nano-DDSs is the fastest growing nano-DDS in the CAS Content Collection (Figure 1B).

Chitosan exhibits mucoadhesive properties and has been used to operate at tight epithelial junctions. Chitosan-based nanomaterials are widely used for sustained drug release systems for various types of epithelia, including intestinal, nasal, buccal, ocular, and pulmonary.^146−150^ Alginate is another biopolymer (polysaccharide) frequently used in drug delivery. Alginate is terminally substituted with carboxylate groups, rendering it anionic and imparting stronger mucoadhesion than that of neutral or cationic mucoadhesive polymers.^151,152^ Xanthan gum is a high MW polyanionic heteropolysaccharide with good bioadhesive properties, produced by *Xanthomonas campestris*. It is widely used as a pharmaceutical excipient since it is considered nontoxic and nonirritating.^153^ Cellulose and its derivatives are extensively used in DDSs mainly for modification of the solubility and gelation of drugs, resulting in control of their release profile.^146−150,153,154^

The combined use of nanotechnology along with the extreme variety of bioactive natural compounds is attractive, and has been growing very rapidly in recent decades.^8^ Natural products have been used as medicines since ancient times. Nowadays, about 35% of the pharmaceutical compounds are either from natural products or their derivatives and analogs, mainly including plant (25%), microbial (13%), and animal (3%) sources.^155^ Natural compounds have been widely studied in curing diseases owing to their various activities, such as inducing tumor-suppressing autophagy and antimicrobial properties. For example, autophagy has been exhibited by curcumin and caffeine,^156^ and antimicrobial effects have been shown by cinnamaldehyde, carvacrol, curcumin, and eugenol.^157,158^ Application of nanotechnologies gave rise to substantial enhancement of their properties, such as bioavailability, targeting, and controlled release. Thus, thymoquinone, a bioactive compound in *Nigella sativa*, exhibited a 6-fold increase in bioavailability after encapsulation in a lipid nanocarrier in comparison to free thymoquinone.^159^ It also improved its pharmacokinetic characteristics, thus accomplishing better therapeutic effects.

### Quantum Dots

Quantum dots are nanometer-sized crystalline semiconductor particles with distinctive fluorescence properties, commonly made of materials such as lead sulfide, lead selenide, cadmium selenide, cadmium sulfide, cadmium telluride, indium arsenide, and indium phosphide.^160−163^ They can also take the form of core–shell structures incorporating two semiconductor materials. They are used primarily in imaging applications and *in vivo* diagnostics.^164,165^ Due to their magnetic, radioactive, or plasmonic properties, these inorganic NPs are particularly suited for applications such as diagnostics, imaging, and photothermal therapies. Most have good biocompatibility and stability and fill niche applications that require properties unattainable by organic materials. For their discovery and development of quantum dots, Bawendi, Brus, and Ekimov were awarded the Nobel prize in Chemistry in 2023.^166,167^ However, quantum dots are limited in their clinical application by low solubility and toxicity concerns, especially in formulations using heavy metals.^88,168^

### Core–Shell NPs

Core–shell NPs are nanostructures in which the core acts as a reservoir for drugs, including small molecules, proteins, nucleic acid therapeutics (DNA, siRNA, or oligonucleotides), or molecular imaging probes, while the shell protects the cargo from the environment.^169−171^ This distinct architecture offers advantages such as tunable physicochemical properties, improved biocompatibility and permeability, target-specific drug delivery, and multidrug delivery. For example, polymer/liposome composite systems with a core/shell structure have been designed, with a lipid vesicle core utilized as a delivery system for small molecules and proteins; NPs with reverse geometry, having polymeric cores, have been also engineered.^169,172,173^ Another intriguing example of core–shell NPs are the spherical nucleic acids^107^ comprising an oligonucleotide shell arranged on an inorganic NP core, mentioned above.

### Biomimetics

Development of NPs with intrinsic characteristics similar to circulatory cells such as leukocytes and platelets for use as biomimetic DDSs has been intended to solve the issues of conventional DDSs. Specifically, synthetic biomimetic NPs coated with cellular membranes have been engineered and shown able to cross the endothelial layer of the inflamed vessels and permeate into tumor tissue mimicking the properties of leukocytes, making it possible to securely deliver drugs to diseased sites.^174^ Thus, biomimetic DDSs, developed by directly utilizing or mimicking biological structures and processes, provide promising approaches for overcoming biological barriers and specifically the blood–brain barrier for brain drug delivery.^175,176^

### Exosomes

Superior innate stability, low immunogenicity, biocompatibility, and excellent capacity for membrane penetration allow exosomes to be valuable natural nanocarriers for efficient drug delivery.^177^ As important mediators of intercellular communications, exosomes are increasingly gaining interest in the context of cancer immunotherapy.^178,179^ Exosomes, either tumor-derived, comprising tumor-associated antigens, or derived from dendritic cells presenting antigens, can trigger immune activation and therefore can be used in developing anticancer vaccines.^180^ Moreover, tumor-derived exosomes hold information from primary cells; thus they can activate CD8 T-cells, which offer distinct therapeutic approaches for developing cancer vaccines.^180−182^ Exosomes participate in the formation of the cancer immunosuppressive microenvironment; thus, tumor exosome production control might be an effective treatment strategy. Exosomes also play a key role in PD-1/PD-L1 immune checkpoint inhibitor treatment.

## Major Material Classes, Top Substances, and Annual Trends

**Figure 2:**
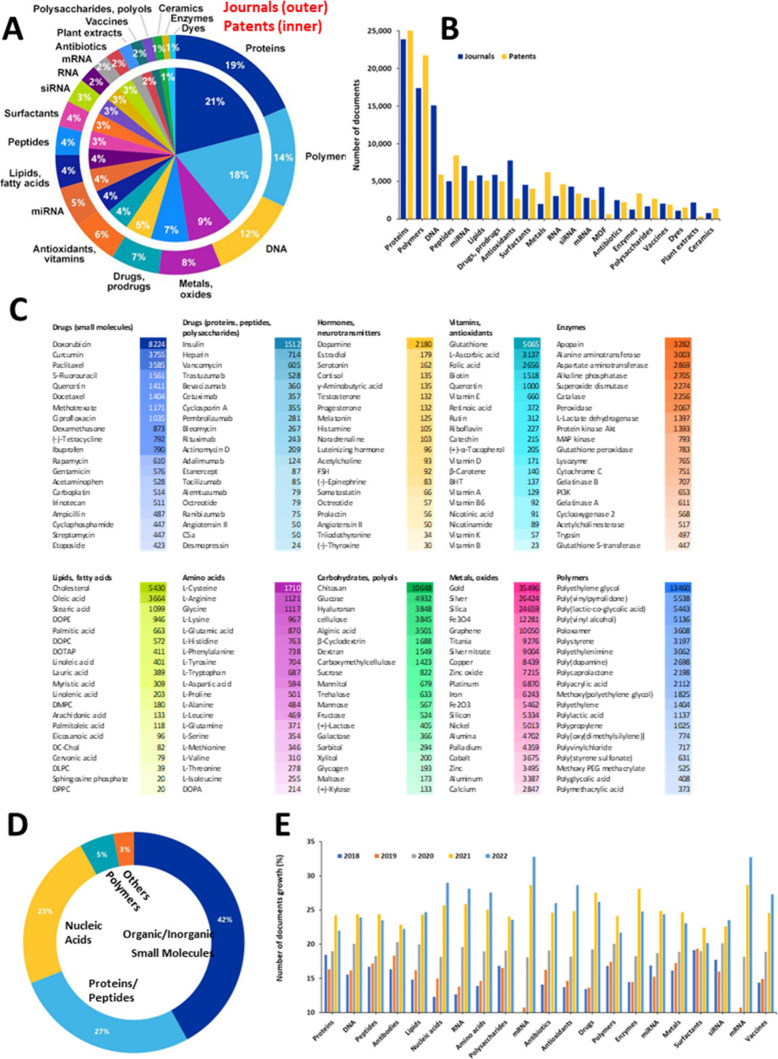
(A) Major substance classes related to nano-DDSs as presented in the CAS Content Collection in the period 2003–2022. (B) Distribution between journal articles (blue) and patents (yellow) for major substance classes. (C) Representative top substances of the major classes related to the nano-DDS. (D) Substance type distribution for the nano-DDS. (E) Publication growth rate of the major substance classes related to the nano-DDS for the 5-year period 2018–2022.

Proteins/peptides are the largest class of substances related to nano-DDSs, both as drugs and as drug carriers (Figure 2A). Natural biomolecules, such as proteins, are commonly used in pharmaceutical nanoformulations because of their safety. Protein nanocarriers offer significant advantages, such as biocompatibility, biodegradability, environmental sustainability, cost efficiency, and availability at larger scales. Furthermore, the preparation procedures and the encapsulation process can be carried out under milder conditions not involving toxic chemicals or organic solvents. Protein nanocarriers can be prepared using various proteins, such as albumin, gelatins, collagens, keratins, silk fibroin, elastin, lipoproteins, and ferritin proteins.^183,184^ Plant proteins such as maize zeins, soy protein, and wheat gliadin are also frequently explored for various drug-delivery applications.^183^

The number of protein/peptide drugs has significantly increased since the introduction of the first recombinant protein therapeutic, which was human insulin.^185,186^ Protein therapeutics have several advantages over small-molecule drugs, such as higher specificity, lower immunogenicity, and faster clinical development and approval. The anticoagulant heparin, the antibiotics vancomycin and bleomycin, along with the antibodies trastuzumab, bevacizumab, cetuximab, pembrolizumab, rituximab, adalimumab, tocilizumab, alemtuzumab, and ranibizumab are the most widely represented protein/peptide drugs in the CAS Content Collection (Figure 2B).

Nucleic acid medicines, including DNA and RNA (miRNA, siRNA, mRNA), have recently shown themselves to be useful in treating a variety of diseases.^187^ However, while nucleic acid therapeutics can expand the range of treatable diseases, their wide-ranging use is limited by multiple delivery challenges.^188^ First of all, nucleic acids need to cross multiple biological membranes, cellular and intracellular, escape from endosomes, and in some cases enter the nucleus. Second, nucleic acids encounter various enzymes upon their delivery to the target cells, which may degrade them or trigger immune response.^189,190^ Third, nonspecific biodistribution to nontarget cells and tissues can lead to low efficacy.^191^ In addition, nucleic acids exhibit a strong negative charge, preventing their permeation across cellular membranes. Thus, delivery vectors for transporting these therapeutics to the desired location are needed.^192^ Beyond the physical barrier of the cellular membrane, there are multiple systemic and intracellular challenges that motivate the need for effective delivery vehicles.^193^ Nucleic acids are subject to endo- and exonucleases that degrade them. Numerous strategies for the encapsulation or stabilization of nucleic acids have been developed in order to achieve intracellular delivery. Common carriers for nucleic acid remedies include cationic polymers, cationic lipids, and cationic peptides.^191^

Polymeric nanocarriers are one of the most widely used nano-DDSs (Figure 1C).^194^ PEG and its copolymers with poly(propylene glycol) (poloxamers), PVP, and polystyrene are among the most common nanocarrier constituents (Figure 2B). Natural polymers such as chitosan, dextrin, polysaccharides, hyaluronic acid, poly(glycolic acid), poly(lactic acid), and their copolymers, have also been widely used for polymeric DDSs. Synthetic polymers such as poly(ethylenimine)s, dendritic polymers, and biodegradable and bioabsorbable polymers have been also discussed for polymeric drug delivery. Cationic polymers form complexes with nucleic acids by means of electrostatic interaction and create a net positive charge of the nanocarriers, which facilitates cell attachment, internalization, and endosomal escape.^195^ The structures of cationic polymers are diverse, including linear polymers such as chitosan and linear poly(ethylenimine), branched polymers such as branched poly(ethylenimine), circle-like polymers such as cyclodextrin, cross-linked poly(amino acids), and dendrimers.^191^

Lipid NPs, one of the widely applied drug nanocarriers, include various lipid constituents, with their compositions determined by the intended morphology and application. Along with the most common constituents, the phospholipids and cholesterol, frequent components of lipid NPs include cationic ionizable lipids and PEG–lipid conjugates (PEG-lipids), as well as various other components.^47^ Cholesterol is the lipid component used in the largest number of nano-DDS-related documents in the CAS Content Collection (Figure 2C). Phospholipids such as phosphatidylcholines, phosphatidylethanolamines, phosphatidylglycerols, and phosphatidylserines, are the most widely used lipid classes. Preferred phospholipid species with respect to their hydrocarbon chains include saturated dimyristoyl, dipalmitoyl, and distearoyl chains, as well as unsaturated dioleoyl chains.^47^ Phospholipids from natural sources, such as soya phospholipids and egg phosphatidylcholines, have also been used often in lipid NP formulations. Since the discovery that PEG–lipid conjugates can significantly increase circulatory half-lives in sterically stabilized “stealth” liposomes, PEG-lipids have also been widely used in pharmaceutical lipid NP formulations.

Cationic lipid NPs, comprising stable complexes between synthetic cationic lipids and anionic nucleic acids, represent the most widely used nonviral delivery system for nucleic acid drugs. Cationic lipids are the most commonly used carriers for nucleic acid delivery. A large number of cationic (ionizable) lipid amphiphiles have been designed, synthesized and tested as nucleic acid carriers since the introduction of *N*-[1-(2,3-dioleoyloxy)propyl]-*N*,*N*,*N*-trimethylammonium (DOTMA).^196^ Commonly used cationic lipids for nucleic acid delivery include various amine derivatives such as DOGS and DC-Chol, quaternary ammonium compounds such as DOTMA, DOTAP, DORIE, and DMRIE, cationic phosphatidylcholines such as EDOPC and EDMPC, combinations of amines such as DOSPA and GAP-DLRIE, and amidinium salts such as Vectamidine.^196−202^ Of particular note are the branched-chain cationic lipids used in the recent mRNA COVID-19 vaccines, ALC-0315 and SM-102.^203−205^

A selection of approved and globally marketed nanotechnology-based drug formulations is summarized in Table 2.

**Table 2:** Globally Marketed Nanotechnology-Based Drug Formulations Approved by Regulatory Agencies such as the US Food and Drug Administration (FDA), the European Medicines Agency (EMA), and Others^8,15,87,184,206−211^

Nano-DDS type	Formulation name	Active ingredient(s)	Company	Indication(s)
**Lipid-based nanomedicine**				
Liposome	DaunoXome^212^	Daunorubicin citrate	Galen	HIV-associated Kaposi’s sarcoma
Liposome	Myocet^213^	Doxorubicin citrate, anthracycline cytotoxic agent	Teva Pharmaceutical Industries	Metastatic breast cancer
Liposome	Visudyne^214^	Verteporfin	QLT PhotoTherapeutics	Severe eye conditions: macular degeneration, decreased vision, ocular histoplasmosis, pathologic myopia
Liposome	DepoDur^215^	Morphine sulfate	Endo Pharmaceuticals	Postoperative analgesia
Liposome	Mepact^216^	Mifamurtide	Takeda France SAS	High grade nonmetastatic osteosarcoma and myosarcoma
Liposome	Lipodox^217^	Doxorubicin hydrochloride	Sun Pharmaceutical Industries (SPIL)	Kaposi’s sarcoma, ovarian cancer, multiple myeloma
Liposome	Lipusu^218^	Paclitaxel	Luye Pharma	Lung squamous cell carcinoma
Liposome	Vyxeos^219^	Daunorubicin and Cytarabine	Jazz Pharmaceuticals	Acute myeloid leukemia
Unilamellar liposome	AmBisome^220^	Amphotericin B	NeXstar Pharmaceuticals	Fungal infections; aspergillosis, candidiasis, cryptococcosis infections
PEGylated liposome	Doxil^221,222^	Doxorubicin hydrochloride	Johnson & Johnson	Ovarian cancer, HIV-associated Kaposi’s sarcoma, multiple myeloma
PEGylated liposome	Caelyx^223^	Doxorubicin hydrochloride	Janssen Pharmaceuticals	Breast cancer, ovarian cancer, AIDS-related Kaposi’s sarcoma
PEGylated liposome	Onivyde^224^	Irinotecan	Merrimack Pharmaceuticals	Metastatic pancreatic cancer
PEGylated cationic lipid NP	mRNA-1273 vaccine^225^	mRNA vaccine	Moderna	COVID-19 infection vaccine
PEGylated cationic lipid NP	Onpattro^226^	Patisiran sodium	Alnylam Pharmaceuticals	Polyneuropathy of hereditary transthyretin-mediated amyloidosis
PEGylated cationic lipid NP	BNT162b2 vaccine^225^	mRNA vaccine	Pfizer	COVID-19 infection vaccine
Pulmonary surfactant	Curosurf^227^	Pulmonary surfactant	Chiesi Farmaceutici	Respiratory Distress Syndrome (RDS)
Nanoemulsion	Diprivan^228^	Propofol	AstraZeneca	Anesthetic agent for sedation of patient under critical carer
Lipid suspension, DMPC & DMPG	Abelcet^229^	Amphotericin B	Liposome Co.	Aspergillosis, invasive fungal infections
Micelle	Apealea^230^	Paclitaxel	Oasmia Pharmaceutical	Ovarian cancer, peritoneal cancer, fallopian tube cancer
**Polymer-based nanomedicine**				
PEGylated protein	Adagen^231^	Adenosine deaminase	Enzon Pharmaceuticals	Adenosine deaminase-severe combined immunodeficiency disorder
PEGylated protein	Oncaspar^232^	l-Asparaginase	Enzon Pharmaceuticals	Acute lymphoblastic leukemia
PEGylated protein	PEGintron^233^	PEGylated interferon α-2B	Merck & Co	Hepatitis
PEGylated protein	Neulasta^234^	Filgrastim	Amgen	Neutropenia
PEGylated protein	Pegasys^235^	PEGylated interferon α-2A	Genentech	Hepatitis B and Hepatitis C
PEGylated protein	Somavert^236^	Recombinant HGH receptor antagonist	Pfizer	Acromegaly
PEGylated protein	Mircera^237^	Epoetin beta	Vifor Pharma	Renal anemia
PEGylated protein	Cimiza^238^	Certolizumab pegol	UCB	Rheumatoid arthritis, Crohn’s disease, psoriatic arthritis, ankylosing spondylitis
PEGylated protein	Krystexxa^239^	Pegloticase	Savient Pharmaceuticals	Severe and treatment-refractory chronic gout
PEGylated protein	Plegridy^240^	Peginterferon β-1a	Biogene	Relapsing remitting multiple sclerosis
PEGylated protein	Adynovate^241^	Recombinant antihemophilic factor	Baxalta US	Hemophilia A
GlycoPEGylated protein	Rebinyn^242^	Recombinant coagulation factor IX	Novo Nordisk	Hemophilia B
Nanoemulsion	Restasis^243^	Cyclosporine	Allergan	Chronic dry eye
Nanoemulsion	Estrasorb^244^	Estradiol hemihydrate	Novavax	Moderate to severe vasomotor symptoms in postmenopausal women
PEGylated aptamer	Macugen^245^	Pegaptanib sodium	Pfizer	Wet age-related macular degeneration
Polymeric micelle	Genexol-PM^246^	Paclitaxel	Lupi	Breast cancer
Polymeric (PLGA) microspheres	Zilretta^247^	Triamcinolone acetonide	Flexion Therapeutics	Knee osteoarthritis
**Nanocrystals**				
Nanocrystal	Avinza^248^	Morphine	King Pharma	Chronic pain
Nanocrystal	Ritalin LA^249^	Methylphenidate hydrochloride	Novartis	Attention deficit hyperactivity disorder in children
Nanocrystal	Zanaflex^250^	Tizanidine hydrochloride	Acorda	Muscle relaxant
Nanocrystal	Emend^251^	Aprepitant	Merck & Co	Antiemetic
Nanocrystal	Tricor^252^	Fenofibric acid	Abott Laboratories	Antihyperlipidemic
Nanocrystal	NanOss^253^	Hydroxyapatite	RTI Surgical	Bone substitute
Nanocrystal	Megace ES^254^	Megestrol acetate	Par Pharmaceuticals	Anorexia, cachexia and AIDS-related weight loss
Nanocrystal	IVEmend^255^	Fosaprepitant dimeglumine	Merck & Co	Antiemetic
Nanocrystal	Focalin XR^256^	Dexmethylphenidate hydrochloride	Novartis	Attention deficit hyperactivity disorder in children
Nanocrystal	Invega^257^	Paliperidone palmitate	Janssen Pharmaceuticals	Schizophrenia
Nanocrystal	Ryanodex^258^	Dantrolene sodium	Eagle Pharmaceuticals	Malignant hypothermia
Nanocrystal	Ostim^259^	Hydroxyapatite	Heraeus Kulzer	Bone substitute
Nanocrystal	EquivaBone^260^	Hydroxyapatite	Zimmer Biomet	Bone substitute
Nanocrystal	Vitoss^261^	Calcium phosphate	Stryker	Bone substitute
Nanocrystal	Rapamune^262^	Sirolimus	Wyeth Pharmaceuticals	Immunosuppressant
**Inorganic NPs**				
Iron NP	DexFerrum^263^	Iron dextran	American Regent	Iron deficiency in chronic kidney disease
Iron NP	Venofer^264^	Iron sucrose	Luitpold Pharmaceuticals	Iron deficiency in chronic kidney disease
Iron NP	Ferrlecit^265^	Sodium ferric gluconate	Sanofi	Iron deficiency anemia
Iron NP	INFed^266^	Iron dextran	Allergan	Iron deficiency anemia
Hafnium oxide NP	Hensify^267^	Hafnium oxide	Nanobiotix	Locally advanced squamous cell carcinoma
Iron oxide NP	Combidex^268^	Iron oxide	AMAG Pharmaceuticals	Magnetic resonance lymphography
Superparamagnetic iron oxide NP	Resovist^269^	Iron oxide	Bayer Schering Pharma	MRI imaging of liver lesions
Gadolinium NP	Primovist^270^	Gadoxetate	Bayer Schering Pharma	MRI imaging of liver lesions
Superparamagnetic iron oxide NP	Endorem^271^	Iron oxide	Guerbet	MRI imaging of liver lesions
Core–shell carbon-dot doped silica NP	C-Dots^272^	Cy5 fluorophore	Elucida Oncology	PET-optical dual-modality imaging
**Protein-based NPs**				
Engineered fusion protein NP	Ontak^273^	Denileukin diftitox	Eisai Co.	Leukemia, T cell lymphoma
Albumin NP	Abraxane^274,275^	Paclitaxel	Eli Lilly	Metastatic breast cancer

## Targeted Diseases and Their Correlation to DDSs

**Figure 3:**
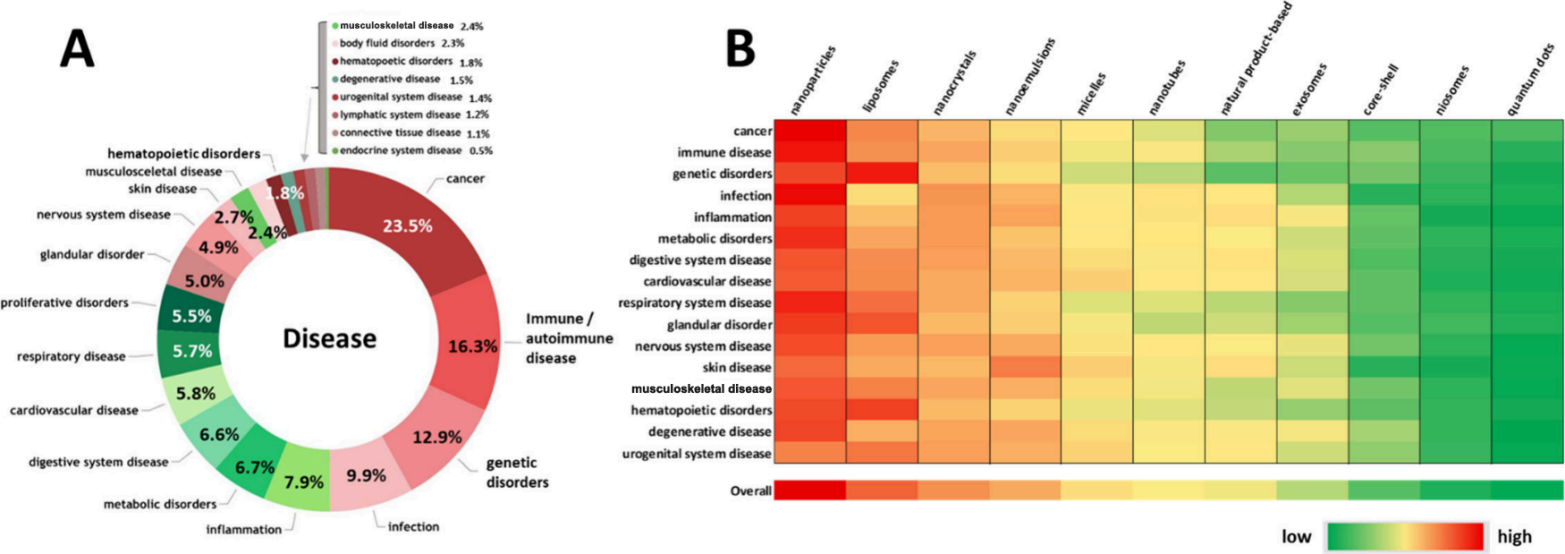
(A) Distribution of nano-DDS-related publications in the CAS Content Collection with respect to targeted diseases. (B) Heat map of the relationship between various types of nano-DDSs and the diseases to which they have been applied.

Development of nanotechnology in nanomedicine is taking place at a rapid pace. The application of nanomaterials ranges from nanosilver for antibacterial use to early diagnosis and treatments of numerous severe diseases such as cancer, immune-related diseases, genetic disorders, infections, inflammation, and many others (Figure 3A). During the last decades, a tremendous amount of research has evaluated diagnostic and therapeutic applications of nanotechnology, some of which have already been approved or have reached advanced clinical trials.

**Cancer** is a major global health threat, causing millions of fatalities yearly.^276^ Nano-DDSs can be engineered to selectively accumulate in tumor tissues, allowing for more precise cancer treatment. Specific targeting of cancer cells is an essential characteristic of nanocarriers for drug delivery, as it offers a way to attack tumors with large doses of drugs, thus augmenting therapeutic efficacy, while protecting normal cells from cytotoxicity and avoiding the harmful side effects that often accompany chemotherapy to the detriment of patients. Passive targeting of nano-DDSs is mainly accomplished by the enhanced permeability and retention (EPR) phenomenon, which exploits the enhanced vascular permeability and weakened lymphatic drainage of cancer cells and enables nanocarriers to target cancer cells passively. Active targeting is attained by interaction between ligands and cellular receptors. Specific receptors on cancer cells include transferrin receptors, folate receptors, glycoproteins (e.g., lectin), and epidermal growth factor receptor (EGFR).^94^ The earliest nanoformulation, approved by the US FDA in 1995, is the anticancer liposomal formulation Doxil, designed to improve the pharmacokinetics and biodistribution of the anthracycline drug doxorubicin.^222^ Multiple other nanomaterial-based pharmaceuticals have received approval and have been successfully used since then for cancer treatment.^222,277,278^

Cancer vaccines, a new cutting-edge approach in immunotherapy, are designed to enhance the immune response against cancer.^279−282^ Nano-DDSs play a crucial role in optimizing these vaccines by improving delivery precision, stability, and the ability to activate immune cells more efficiently. Gardasil and Cervarix are both vaccines that protect against human papillomavirus (HPV) types 16 and 18, common sexually transmitted infectious agents, which are responsible for most HPV-caused cancers such as cervical, anal, throat, and other cancers.^283,284^ Hepatitis B vaccine prevents liver cancer, which can be caused by hepatitis B virus (HBV). This preventive vaccine protects against HBV infection, thereby lowering the risk of chronic liver disease and hepatocellular carcinoma.^283^ Provenge (Sipuleucel-T) is a therapeutic cancer vaccine that uses the patient’s own dendritic cells and was approved in 2010 for castration-resistant prostate cancer.^280^ mRNA cancer vaccines are personalized to each individual based on their tumor’s molecular features.^285,286^ They work by training the immune system to recognize and fight cancer cells. Clinical trials are testing mRNA vaccines for various types of cancers including pancreatic cancer, colorectal cancer, melanoma, and advanced head and neck cancer. ECI-006 is a combination mRNA cancer vaccine that combines mRNA encoding dendritic cell activation molecules with mRNA encoding tumor-associated antigens.^287^ Other promising cancer vaccines include adoptive T-cell transfer and allogeneic whole cell vaccines.^288,289^

Another complementary nanotechnology-based approach for the treatment of cancer is therapeutic hyperthermia, a technique in which the body temperature is locally raised above the normal level.^290,291^ The response of cancer cells to radiation and chemotherapy can be augmented by increasing the temperature within tumors.^290^ NPs are applied to induce localized heating within tumors. Hyperthermia can be induced by either laser radiation or an applied magnetic field. Magnetic NPs can be used as heating mediators.^292^ The specific optical properties of noble metal NPs have been used for inventive light-based treatment approaches for cancer treatment. Thus, the combination of noble metal (Au, Ag) and magnetic iron oxide NPs is reported to augment the effectiveness of hyperthermia.^290,291,293^ The response of cancer cells to radiation and chemotherapy can be augmented by increasing the tumor temperature.^290^

Numerous diseases have their sources at the **genetic** level. The human genome project and the advances in molecular genetics and high throughput technologies have revealed the genetic basis of many pathologies and identified new therapeutic approaches. Gene-based therapies must cross multiple biological barriers in order to reach their sites of action. Because of their negative charge, nucleic acids cannot cross the cellular membrane, which is also negatively charged. Therefore, delivery vehicles that allow gene medicines to reach their site of action avoiding degradation, crossing cellular membranes, and escaping the endosomes, are needed.^294^ Nanomaterials are presently being developed for the delivery of genetic material, as nonviral vectors for gene therapy use. A number of nanostructures including lipids, polymers, and various inorganic nanocarriers can incorporate certain genetic materials, such as plasmid DNA, mRNA, and siRNA. One of the most significant applications for nanomaterial-based gene delivery is the use of NPs in genetic-based vaccines.^295,296^

**Infectious diseases** are a dominant driver of global disease burden. High mortality rates are associated with lower respiratory infections, diarrhea, tuberculosis, human immunodeficiency virus (HIV) infection, and malaria.^297^ Nanotechnology-based approaches have been the focus of intensive research efforts to improve the therapeutic index of anti-infective drugs and simplify their use. The introduction and advancement of medical nanotechnology can develop a more straightforward treatment regimen with a lower dose frequency. Long-acting injectable NPs comprising antiretroviral drugs are an emerging treatment method for reducing the frequency of doses for HIV patients and represent the most clinically advanced nanotechnology treatment for this virus. Nanotechnology like this also has the potential to be used as a preventative measure, which could benefit a large population who are at a higher risk for HIV. The targeting potential of nanotechnology is a significant advantage, helpful in overcoming challenges associated with the treatment of these diseases, including low on-target bioavailability and low patient adherence due to drug-related toxicities and extended therapeutic regimens.^298^ It would be significantly beneficial for treatment of malaria, usually treated with chemotherapy drugs that have adverse side effects and suffering from toxicity, missed doses, and the development of resistance. Furthermore, nanocarriers can be applied for formulating vaccines, which represent a major defense in combating infectious diseases^298,299^

**Antibiotic drug resistance** has been identified as a global concern by the World Health Organization since 2014^300,301^ and is still regarded as a primary health concern.^302^ A major contributing factor to the rise of multidrug resistance (MDR) is the rampant misuse of antibiotics in both humans and animals (as part of the food industry).^210,303^ This, along with the slow pace of development of additional antibiotics, has further intensified the MDR crisis. In this context, the exploration of other avenues, such as use of nano-DDSs to combat MDR, has become a vital need. Furthermore, repurposing known classes of antibiotics into nanomaterial-based DDSs has been found to overcome resistance mechanisms and can potentially help reduce the burden of MDR.^304,305^

The present treatments for **autoimmune diseases** involve administration of broad-spectrum, nonspecific, anti-inflammatory, or immunosuppressive drugs, which reduce the proliferation of inflammatory cells and inhibit immune reactions. Such treatment can alleviate clinical symptoms but is unable to address the underlying cause and therefore incapable of curing the disease. Moreover, extensive use of immunosuppressants reduces the body’s normal immune response, increasing susceptibility to other diseases.^306,307^ The application of nanocarrier-based DDSs in the treatment of autoimmune diseases such as rheumatoid arthritis, multiple sclerosis, and lupus can increase the efficiency of inducing antigen-specific tolerance *in vivo*.^307^ Nanocarriers have significant potential as tolerance delivery vehicles with certain benefits to autoimmune diseases, allergies, and transplantation rejection immunotherapy. Nanocarrier-mediated delivery-induced tolerance *in vivo* is a promising approach in autoimmune diseases or transplantation. The capability of NPs to deliver antigens and immunomodulators, primarily targeting antigen-presenting cells and lymphocytes, can increase the potential to induce a specific tolerance.

**Inflammation**, a common feature of numerous diseases, is a basic immune response that facilitates survival and sustains tissue homeostasis. In some conditions, the inflammatory process becomes harmful, contributing to the pathogenesis of a disease. Targeting inflammation by using nanomedicines, either through the detection of molecules overexpressed on the surface of activated macrophages or endothelial cells or via enhanced blood vessel permeability, provides a promising solution for the treatment of inflammatory diseases.^308^ Various types of nanocarriers have been developed or are still in development for the management of inflammation, including liposomes, polymer NPs, micelles, dendrimers, or hydrogel-based formulations, which can target passively, through the leaky vasculature, or actively the main triggers of inflammation, including macrophages, endothelial cells, membrane receptors on inflammatory cells, anti-inflammatory genes, and cytokines.^308^

The various types of nano-DDSs and the diseases with which they co-occur in the CAS Content Collection are depicted in Figure 3B as a heat map. In most diseases, NPs are the most frequently used nano-DDS.

For **genetic disorders**, liposomes are the preferred delivery systems. Indeed, after the invention of cationic lipids in 1987,^196^ cationic liposomes have been widely applied for gene delivery.^197,309^ Complexation with positively charged lipids stabilizes nucleic acids and enhances their resistance to nuclease degradation, allowing them to be delivered to their desired target cells.

Liposomes are the preferred nano-DDS for treating hematopoietic disorders, as well (Figure 3B). Introduced in the 1990s, PEGylated liposomal doxorubicin has been approved as an antitumor agent in the US and other countries and is widely used in patients with multiple myeloma.^310^ Another liposome-encapsulated formulation delivers a synergistic 5:1 drug ratio of cytarabine and daunorubicin for treating acute myelogenous leukemia.^311^ It was recently reported that surface-modified liposomes can present a promising approach to deliver liposomal drugs into bone marrow via specific bone marrow phagocytosis.^312^ This bone marrow delivery formulation can be a successful nanocarrier for the therapy of hematopoietic malignancies such as myelocytic leukemia and multiple myeloma.

One of the larger uses of liposomal DDSs is for treating urogenital diseases (Figure 3B). It was recently reported that intravesical instillation of liposome-encapsulated botulinum toxin A can be a successful treatment for functional bladder disorders such as overactive bladder, interstitial cystitis/bladder pain syndrome, and bladder oversensitivity.^313^ Liposomal tacrolimus instillations have been reported to be promising for the treatment of hemorrhagic cystitis.^314,315^ Liposomal amphotericin B has been found effective in the treatment of urinary tract infections caused by *Candida albicans*.^316^

Nano-DDSs have shown great promise in the field of **wound healing**, as they can enhance the effectiveness of treatments, promote tissue regeneration, and reduce infection risks.^317−326^ By using NPs to deliver drugs, growth factors, or other therapeutic agents directly to the wound site, these systems aim to improve the healing process. Antimicrobial NPs prevent infection by delivering antimicrobial agents directly to the wound. For example, silver NPs can be used to combat a wide range of bacteria, fungi, and viruses. They can be incorporated into wound dressings, gels, or sprays to prevent infection in chronic wounds, burns, and diabetic ulcers. Zinc oxide NPs also exhibit strong antibacterial activity and can be used in wound dressings. Growth factor delivery promotes tissue regeneration and speeds up the healing process by delivering growth factors that stimulate cell proliferation and tissue repair. Polymeric NPs encapsulating growth factors, such as VEGF or PDGF can be used to enhance angiogenesis. Chitosan NPs can be used to deliver growth factors to wounds and stimulate faster healing. Anti-inflammatory NPs reduce inflammation in chronic or nonhealing wounds. Curcumin-loaded NPs help to reduce inflammation and promote faster wound healing. Dexamethasone-loaded NPs can be delivered to control excessive inflammation in chronic wounds or burns. Collagen-based NPs support tissue regeneration by delivering collagen, which is a key structural protein in the skin. Nanofibers and nanogels serve as wound dressings that not only protect the wound but also release therapeutic agents over time. NPs can enhance blood flow to the wound by promoting angiogenesis, which is critical for supplying nutrients and oxygen to the regenerating tissue.^317−326^

Topical delivery of active pharmacological ingredients is a challenge, because of the mechanical barrier created by the skin. Nanoemulsions have emerged as a promising nano-DDS in the field of dermatology for the encapsulation of active substances and for their controlled release. Indeed, decreasing particle size in nanoemulsions increased the contact surface area, resulting in increased drug efficacy and generally exhibiting superior performance in safety, permeability, and bioavailability.^327^ Polymeric micelles are another successful topical nanocarrier. They have been reported to enhance the deposition of drugs in targeted sites of the skin in dermatological diseases such as psoriasis and acne.^328^

Exosomes secreted by cells involved in inflammation exhibit high inflammatory affinity and targeting; hence they can successfully deliver cargo to inflammatory cells and can achieve superior anti-inflammatory effect.^329^ Exosomes derived from mesenchymal stem cells, astrocytes, and dendritic cells with immunomodulatory functions are widely applied as delivery vehicles to transport cargo to inflammatory sites for enhanced anti-inflammatory efficiency.^329−331^ Successful application of exosomes has been also reported in a variety of conditions, including neurodegenerative diseases,^332^ cardiovascular diseases,^333^ and cerebrovascular diseases,^334^ and others.^329,335^

## Delivery Routes and Their Correlation with DDS Types

**Figure 4:**
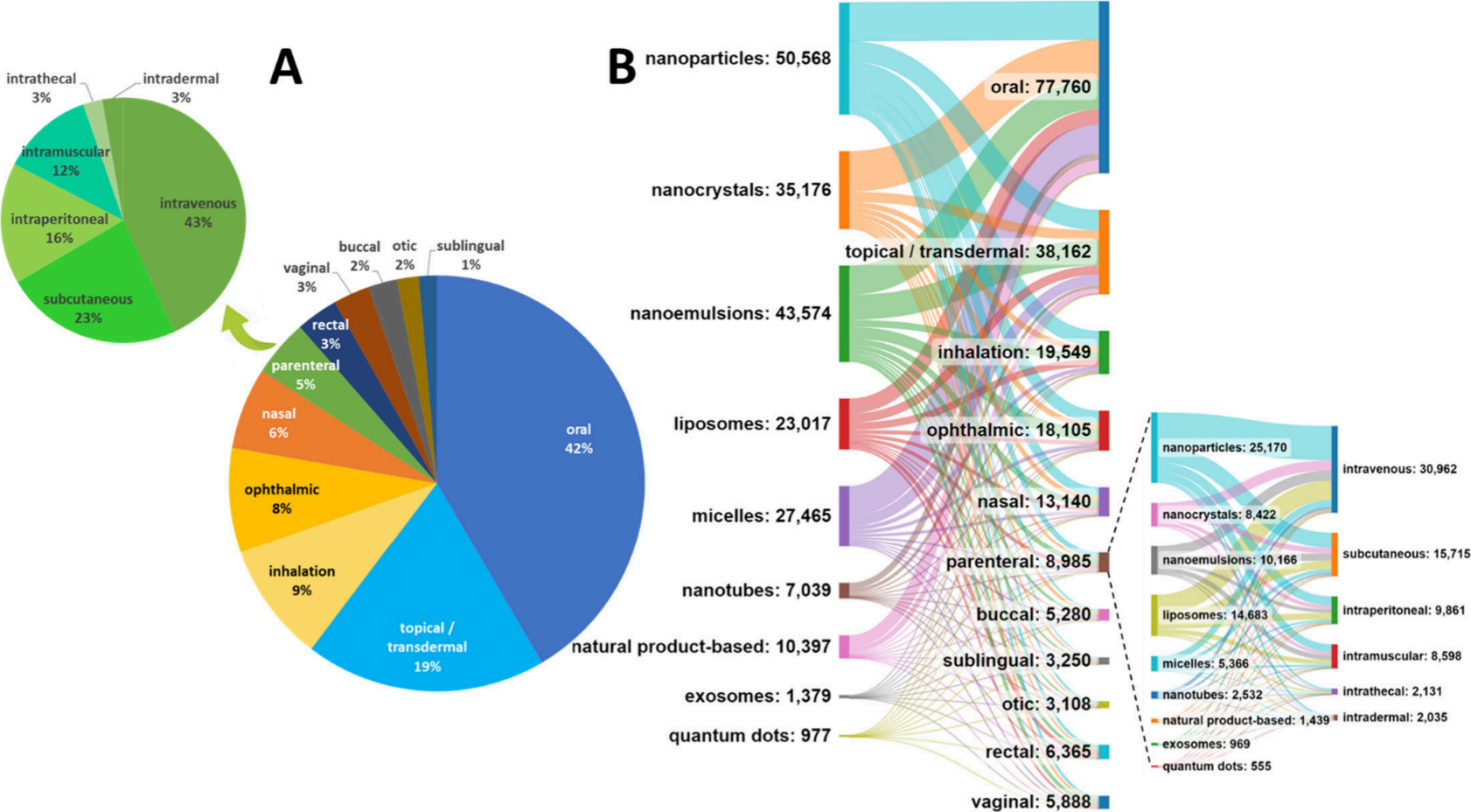
(A) Distribution of documents in the CAS Content Collection related to various administration routes of the nano-DDSs. (B) Sankey diagram depicting co-occurrences between the type of nano-DDS and its administration routes.

Nanomedicines can be administered through various routes depending on the specific characteristics of the nanomaterials and the targeted disease (Figure 4). The choice of administration route is influenced by factors such as the desired therapeutic effect, the site of action, and the physical and chemical properties of the nanomedicine.

NPs can be incorporated into **oral** formulations such as tablets, capsules, and liquid suspensions. The oral route is a preferred method of drug administration owing largely to its facility, convenience, and highest degree of patient compliance; however effective drug delivery with minimum off-target side effects is often challenging.^336^ NPs can protect drugs from degradation in the digestive system and enhance their absorption. Formulation into NPs can enhance drug stability in the harsh gastrointestinal tract environment, improving the likelihood for successful targeting, increasing drug solubility and bioavailability, and affording sustained release within the gastrointestinal tract.

NPs can be delivered directly into the bloodstream through **intravenous injection**. This route is commonly used for the systemic delivery of nanomedicines to target specific organs or tissues throughout the body. Injection of nanomedicines into muscle tissue (**intramuscular**) or just beneath the skin (**subcutaneous**) allows for sustained release and gradual absorption. This route is often used for the sustained delivery of drugs or vaccines. Injection of nanomedicines directly into the peritoneal cavity (**intraperitoneal**) or the tumor site (**intratumoral**) can be used for localized treatments. This route is often employed in cancer therapy to deliver drugs directly to the tumor. Injection of nanomedicines into cerebrospinal fluid (**intrathecal**) or directly into brain tissue (**intracerebral**) can be used for treating neurological disorders. This route allows for bypassing the blood–brain barrier to deliver therapeutic agents to the central nervous system. Injection of nanomedicines into the skin (**intradermal**) or delivery through the skin (**transdermal**) is employed for localized treatments or sustained drug release. Transdermal patches containing NPs can facilitate controlled drug delivery over an extended period.

NPs can be administered directly into the vitreous humor of the eye (**intravitreal**) for the treatment of ocular diseases. This route is used to target specific tissues within the eye while minimizing systemic exposure. NPs can be engineered for **inhalation**, allowing for targeted delivery to the respiratory system. This route is useful for treating lung diseases and achieving the rapid absorption of drugs into the bloodstream through the lungs. NPs can be formulated for **nasal** delivery, providing a noninvasive route for systemic or local drug delivery. This route is particularly advantageous for drugs that may be degraded in the digestive system.

The selection of a specific delivery route depends on the therapeutic goals, nature of the drug, and characteristics of the targeted disease or condition. Each route comes with its own set of considerations, advantages and disadvantages (Table 3), and ongoing research aims to optimize drug delivery for improved efficacy and patient outcomes.

**Table 3:** Advantages and Disadvantages of the Administration Routes of nano-DDSs^337−343^

Administration route	Advantages	Disadvantages
Intravenous (iv)	Provides direct access to the bloodstream, ensuring a rapid onset of action.	Invasive method which requires a skilled healthcare professional.
	Avoidance of first-pass metabolism results in high bioavailability.	Potential for infection at the injection site.
	Precise dosing due to direct delivery into the systemic circulation.	May cause undesirable immune reaction.
Oral	Noninvasive.	Subject to first-pass metabolism, reducing bioavailability.
	Convenient and promotes better patient compliance.	Absorption can be inconsistent due to factors such as gastrointestinal pH and enzymatic activity.
	Cost effective.	
Transdermal	Noninvasive.	Limited permeability.
	Allows for sustained and controlled release over an extended period.	May cause enzymatic deterioration.
	Prevents deterioration of drug due to gastrointestinal interaction.	
Inhalation	Direct pulmonary delivery.	Ensuring optimal particle size for deep lung penetration can be challenging.
	Quick absorption, due to the large surface area of the lungs.	May cause irritation in the respiratory tract.
Intramuscular (im)/Subcutaneous (sc)	Allows for controlled release, especially with sustained-release formulations.	Requires a healthcare professional for administration.
	Bypasses first-pass metabolism to some extent, enhancing bioavailability.	May cause local reactions at the injection site.
Intraperitoneal (ip)	The peritoneal cavity provides a large surface area for drug absorption.	Requires a skilled healthcare professional for administration.
	Bypasses first-pass metabolism, leading to increased bioavailability.	Potential for infection at the injection site.
Intranasal	Noninvasive.	Restricted to small drug volumes due to nasal cavity constraints.
	Rapid absorption due to the rich blood supply in the nasal mucosa.	Absorption may vary among individuals.
	Prevents interaction with gastrointestinal tract.	Intolerance in nasal mucosa.
Intrathecal/Intraventricular	Direct drug delivery to the cerebrospinal fluid (CSF) for CNS disorders.	Invasive, involves injection into the spinal canal or brain ventricles, requiring expertise.
	Bypasses the blood–brain barrier, enhancing drug access to the CNS.	Carries a risk of infection and potential neurological complications.
Rectal	Bypasses first-pass metabolism, improving bioavailability.	Absorption may be variable and dependent on rectal conditions.
	Absence of enzymes helps in avoiding enzymatic degradation.	Patient acceptance may be lower due to the nature of administration route.
	Administered rectally, offering a noninvasive alternative.	
Ocular	Allows for targeted drug delivery to the eyes for ocular conditions.	Limited volume capacity in the eye for drug administration.
	Minimizes systemic exposure, reducing potential side effects.	Some formulations may cause eye irritation.
Vaginal	Targeted delivery for gynecological conditions, minimizing systemic exposure.	Absorption may vary among individuals.
	Bypasses first-pass metabolism for improved bioavailability.	May cause local irritation in the vaginal mucosa.

## Applications: Therapy, Diagnostics, Imaging, Cosmetics, Nutraceuticals, and Agriculture

DDSs have found diverse applications beyond the traditional medical field, including drug/vaccine/gene delivery and diagnostic/imaging, extending into areas such as food and dietary supplements, cosmetics, agriculture, and others.

In the field of **food and dietary supplements**, NPs and microencapsulation technologies are applied to protect sensitive nutrients, such as vitamins and omega-3 fatty acids, from degradation, ensuring their stability and bioavailability. Encapsulation can also be used to mask undesirable tastes or aromas, protecting sensitive flavors, or adding controlled-release properties to enhance the sensory experience of food and dietary supplements. Microencapsulation helps protect probiotics from harsh stomach conditions, ensuring their survival and efficacy in the digestive system. Nanodelivery systems enhance the solubility and absorption of poorly soluble nutrients such as vitamins (e.g., vitamin D and vitamin E), minerals, omega-3 fatty acids, and antioxidants. For example, lipid NPs (e.g., nanoemulsions or nanoliposomes) are used to encapsulate fat-soluble vitamins and enhance their absorption in the gastrointestinal tract. NPs protect sensitive nutrients from degradation due to exposure to light, oxygen, or heat during processing or storage. Thus, nanoencapsulation of probiotics in dairy or nondairy products protect them from stomach acid and ensure that they reach the intestine intact. Nanodelivery systems can also provide controlled or sustained release of nutrients to ensure that they are absorbed gradually, maximizing their health benefits.^344−350^

DDSs in **cosmetics** involve the encapsulation of active ingredients in nanocarriers such as liposomes or NPs. This ensures controlled release and targeted delivery into the skin for improved efficacy. NPs improve the penetration of active ingredients into deeper layers of the skin, making cosmetics and skin care products more effective. For example, lipid-based NPs like nanoliposomes or SLNs are used in antiaging creams to deliver ingredients such as retinol, peptides, or coenzyme Q10 deep into the skin. NPs can be used to deliver sun-blocking agents, improving the stability and distribution of sunscreens on the skin. Nanodelivery systems can release skin care or cosmetic ingredients in a controlled manner, providing long-lasting effects. Thus, nanospheres loaded with moisturizers or sunscreens release ingredients slowly throughout the day, providing prolonged hydration or UV protection.^351−357^

In **agriculture**, NPs and microencapsulation are utilized for the controlled release of fertilizers, pesticides, and growth regulators. This promotes precision farming, reduces environmental impact, and enhances the crop yield. Controlled-release systems in agriculture involve encapsulating fertilizers in polymer coatings, allowing for gradual and sustained release of nutrients to crops. Nanocarriers can be used for the targeted delivery of biopesticides, minimizing the environmental impact of pest control. Nanocapsules encapsulating pesticides or herbicides allow for controlled release at specific times or in response to environmental triggers such as humidity or pH changes in the soil. Nanofertilizers made from polymeric NPs deliver micronutrients such as nitrogen, phosphorus, or potassium directly to plant roots, increasing efficiency. NPs can be used to deliver growth stimulants, plant hormones, or genetic materials to enhance crop growth and resilience. For example, NPs delivering plant growth regulators such as gibberellic acid can improve seed germination, flowering, and fruit development.^358−362^

Figure S6 shows the percentage of documents (journal articles and patents) related to the various application fields, as well as their relative annual growth. As anticipated, the medical applications including drug/vaccine/gene delivery and diagnostic/imaging dominate, comprising in combination 91% of journal articles and 82% of patents.

## Notable Patents

In recent years, sizable methodological progress and a wealth of knowledge have promoted the advancement of research on nano-DDSs, enhancing our understanding of their structure and efficiency. This is reflected in the consistent growth in the number of related scientific publications (journal articles and patents) in the last two decades. The landscape of the nano-DDSs research as reflected in the CAS Content Collection is presented briefly in the Supporting Information, Figures S1–S6.

Table 4 summarizes exemplary notable patents related to nano-DDSs. These examples were selected to represent the range of discussed materials and applications and also based on innovative uses of nanomaterials in DDSs.

**Table 4:** Notable Patent Application Publications in the Field of Nano-DDSs in Recent Years

Patent Number	Publication Year	Patent Assignee	Title	Details
WO2023237788	2023	Cellvie (Switzerland)	Mitochondria as a targeted delivery platform	A mitochondrion comprising payloads including nucleic acids, polypeptides, drugs or a combination thereof, electrostatically attached to the outer membrane of the mitochondrion, to provide a drug delivery platform of notable efficiency
WO2020061367	2020	ModernaTX (USA)	Compounds and compositions for intracellular delivery of therapeutic agents	Preparation of novel lipids and their NP compositions useful in delivery of therapeutic and/or prophylactic treatments such as RNA, with improved endosomal escape and sustained efficiency and safety
WO2023144127	2023	AGS Therapeutics (France)	Extracellular vesicles from microalgae, their biodistribution upon administration, and uses	DDSs containing extracellular vesicles from microalgae loaded with bioactive cargo, administered by a variety of routes, with applications as therapeutics, including as vaccines, as anticancer therapeutics, and as therapeutics for psychiatric diseases
US20120040397	2012	Cornell University (USA)	Photo-cross-linked nucleic acid hydrogels	Methods and compositions for producing hydrogel nucleic acid structures using photo-cross-linking, and using these hydrogels for cell-free protein production, and for encapsulating and delivering compounds
WO2013086373	2013	Alnylam Pharmaceuticals (USA)	Lipids for the delivery of nucleic acids	Novel cationic lipids that can be used in combination with other lipid components such as cholesterol and PEG-lipids to form lipid NPs with oligonucleotides, to facilitate the cellular uptake and endosomal escape, and to knockdown target mRNA
WO2021077066; WO2021077067	2021	University of Pennsylvania (USA)	Lipid nanoparticles and formulations thereof for CAR mRNA delivery	Lipid NPs for delivery of mRNAs encoding chimeric antigenic receptor (CAR), nucleic acid, and/or therapeutic agents to selected target cells
WO2011076807	2011	Novartis (Switzerland)	Preparation of cationic and stealth lipids and compositions for drug delivery	Compositions comprising cationic lipids, stealth lipids, and helper lipids and optimization protocols for delivery of therapeutically effective amounts of active agents to liver, tumors, and/or other cells or tissues
WO2021030776	2021	Codiak Biosciences (USA)	Extracellular vesicle-antisense oligonucleotide constructs targeting STAT6 and use for treating disease	Exosomes, comprising an antisense oligonucleotide with a contiguous nucleotide sequence complementary to a nucleic acid sequence within a STAT6 transcript, as well as methods for producing the exosomes and using them to treat and/or prevent diseases
US20060040286	2006	Nanosphere (USA)	Utilizing reporter oligonucleotides as bio-barcodes for detection of target analytes and diagnostic uses	Screening methods and kits for detecting the presence or absence of one or more target analytes, e.g., proteins, such as antibodies, nucleic acids, or other compounds in a sample; in particular, reporter oligonucleotides are used as biochemical barcodes for detecting multiple protein structures in a solution
WO2018227012	2018	Massachusetts Institute of Technology (USA)	Polymer–lipid materials for delivery of nucleic acids	NPs comprising a conjugated polyethylenimine polymer (conjugated lipomer) and a lipid–PEG conjugate, useful for the delivery of active agents, for the treatment of disease
WO2022159855	2022	Johns Hopkins University (USA)	Photo-cross-linked bioreducible polymeric nanoparticles for enhanced RNA delivery	Photo-cross-linked bioreducible NPs for stable siRNA encapsulation in high serum conditions, shielded surface charge, efficient intracellular trafficking, and triggered cytosolic RNA release, allowing robust siRNA-mediated knockdown in cancer cells and systemic siRNA delivery to tumors in lungs
WO2021119402	2021	Harvard College (USA)	Compositions and methods for light-directed biomolecular barcoding	Compositions and methods for nucleic acid barcoding that can be used to linearly, combinatorially, or spatially barcode a plurality of targets in a sample, as well as a device for use in a barcoding method comprising a light source and a sample holder
WO2023092040	2023	Northwestern University (USA)	Spherical nucleic acids for cgas-sting and stat3 pathway modulation for the immunotherapeutic treatment of cancer	Spherical nucleic acids: nanostructures comprising a NP core and a shell of oligonucleotides attached to the external surface of the NP core, the oligonucleotide shell comprising a double-stranded or single-stranded stem–loop DNA oligonucleotide activating cyclic GMP–AMP synthase
WO2012110636	2012	Instituto Nacional de Investigacion y Tecnologia Agraria y Alimentaria (Spain)	Carrier peptides for cell delivery	Delivery of molecules into cells, using peptide binding proteins from the cell microtubule motor complex, preferably dynein-binding peptides, as carrier/delivery peptides; or functionalized structures, such as NPs, linked to said peptides, for use in diagnosis, therapy, and pharmacology
WO2005116226	2005	Midatech; Consejo Superior de Investigaciones Cientificas (Spain)	Magnetic nanoparticles comprising metals and semiconductor atoms conjugated to siRNA or microRNA for diagnosis and therapy of diseases	Magnetic NPs having a core comprising metals and semiconductor atoms conjugated to siRNA or microRNA for diagnosis and therapy of diseases, for targeted transcriptional gene silencing, for targeted mRNA degradation, for imaging mRNA, as a tool in functional genomics

## Outlook, Challenges, and Perspectives

The application of nanotechnology in biomedical sciences, in healthcare as a whole, and specifically in drug delivery is considered an emerging area of nanotechnology, playing a significant role in the field of medicine and pharmaceutics, mainly due to its potential to overcome the major limitations and problems related to conventional DDSs. The outlook for nano-DDSs is promising, with ongoing research addressing challenges and paving the way for innovative and impactful therapeutic solutions. The major perspectives and expectations for nano-DDS can be summarized as follows:

- Personalized treatment paradigm: Nano-DDSs contribute to the growth of precision medicine by enabling targeted and personalized therapies. They offer the potential to shift toward more patient-centric treatment approaches. For example, lipid NPs are used to encapsulate mRNA that encodes tumor antigens, ensuring that the mRNA is delivered to cells where it can produce the antigen and stimulate an immune response. This technology is being explored in personalized cancer vaccines. The synergy between nano-DDSs and cancer vaccines holds great promise for creating more effective and personalized cancer therapies.
- Therapeutic innovation: Due to their customizable structure and surface modifications, they facilitate the delivery of a wide range of therapeutic agents, including small molecules, biologics (like proteins and antibodies), and nucleic acids (like DNA and RNA), allowing for targeted delivery to specific sites within the body while potentially improving treatment efficacy and reducing side effects.
- Multifunctional platforms: NPs can carry multiple therapeutic agents (e.g., a drug and an imaging agent), allowing for combination therapy and real-time monitoring of treatment. For example, theranostic NPs combine therapeutic and diagnostic capabilities, enabling simultaneous treatment and imaging of tumors.
- Disease-specific approaches: Nanocarriers can be tailored for specific diseases by utilizing distinct surface properties that allow them to selectively deliver drugs to diseased cells, thereby enhancing treatment efficacy while minimizing side effects compared to traditional drug delivery methods. NPs can be engineered with specific ligands or antibodies that bind to receptors overexpressed on diseased cells, ensuring that the drug reaches the intended target tissue. By delivering drugs directly to the target site, healthy tissues are exposed to less medication, minimizing systemic side effects.
- Combination therapies: They provide opportunities for combining multiple drugs in a single nanocarrier allowing for the controlled delivery of different therapeutic agents simultaneously, potentially leading to enhanced synergistic effects between the drugs, meaning their combined effect is greater than the sum of their individual effects when administered separately. However, designing stable nanocarriers that can effectively encapsulate and deliver multiple drugs with different physicochemical properties can be challenging.
- Overcoming biological barriers: Ongoing research focuses on designing NPs to overcome biological barriers, such as the blood–brain barrier, which are typically difficult for conventional drugs to penetrate. For example, lipid-based NPs or dendrimers can be used to deliver drugs directly to the brain, offering new therapeutic options for neurodegenerative diseases and brain tumors.
- Remote-controlled delivery: Advancements are made in remote-controlled or stimuli-responsive nanosystems for on-demand drug release. Nanoassemblies responsive to exogenous stimuli are employed for remotely controlled drug delivery. The properties of the nanoassembly based DDSs are dependent on stimuli-triggered structural transition. Such nanoassemblies hold great potential for clinical translation.
- Drug repurposing opportunities: Nanocarriers provide opportunities for repurposing existing drugs by improving their delivery and efficacy. Using NPs as drug carriers introduces fresh opportunities for drugs that exhibit potent therapeutic properties, but are hampered by delivery-related complications. The extensive range of attributes and adaptability associated with NPs renders them particularly interesting for drug repurposing.
- Global health impact: Nanomedicine has rapidly grown to treat certain diseases like brain cancer, lung cancer, breast cancer, cardiovascular diseases, and many others. These nanomedicines can improve drug bioavailability and drug absorption time, reduce release time, eliminate drug aggregation, and enhance drug solubility in the blood. Addressing these challenges can lead to breakthroughs that impact global health, especially in the treatment of complex diseases.
- Advancing cancer treatment: Nano-delivery systems continue to play a crucial role in advancing cancer treatment options, providing targeted and less toxic alternatives: NPs can selectively target tumor tissues, sparing healthy cells; they are more stable and biocompatible than conventional drugs; they can accumulate in tumors due to defects in the tumor microenvironment; the timing and site of drug release can be controlled using external or internal triggers; they can target mechanisms that cause cancer drug resistance.
- Emerging applications: Nano-DDSs can be utilized in new applications, such as in the delivery of gene-editing tools and RNA-based therapies. Implant technology can deliver targeted medication for longer-lasting, localized therapy.
- Regulatory adaptations: There are ongoing efforts to adapt regulatory frameworks to accommodate the specific characteristics of nano-DDSs. Regulatory agencies worldwide are actively adapting their frameworks to address the distinct characteristics of nano-DDSs, focusing on establishing specific guidelines for evaluating the safety and efficacy of nanomaterials used in drug delivery, including detailed characterization requirements, toxicity testing protocols, and considerations for targeted delivery mechanisms, to ensure the safe and effective translation of nanomedicines from research to clinical applications.

Along with the benefits, nano-DDSs need to address certain challenges and roadblocks that impact their development, translation to clinical use, and widespread application. These include the following:

- Biocompatibility and toxicity: the potential toxicity of nanomaterials, especially inorganic NPs like gold or silver, raises concerns. While these materials are effective for drug delivery, they can accumulate in organs such as the liver, spleen, or kidneys, leading to long-term toxic effects. Determining safe dosages and long-term biocompatibility is critical. Currently, there are limited studies on the long-term impacts of nanomaterials on human health, particularly in terms of chronic toxicity and biodegradability.
- Clinical translation: complex manufacturing processes, concerns about toxicity and biocompatibility, difficulty in achieving targeted delivery, lack of standardized regulatory pathways, high production costs, and the need to demonstrate significant clinical benefits compared to existing treatments all can significantly hinder the progress from preclinical studies to clinical trials and market approval.
- Scale-up challenges: Manufacturing NPs on a large scale with consistent quality and safety is a major hurdle. Scaling up nanoformulations from laboratory to industrial production requires strict quality control to ensure uniformity in particle size, drug encapsulation efficiency, and release properties. The production process of nanocarriers is highly sensitive to variations in conditions, such as temperature, solvent use, and particle synthesis methods. Small changes can affect the properties of NPs, making reproducibility difficult. The cost of producing NPs at an industrial scale also remains high, posing a barrier to widespread adoption.
- Biodistribution variability: even under normal physiological conditions, effective biodistribution and drug delivery are difficult to achieve as NPs face both physical and biological barriers, including shear forces, protein adsorption and rapid clearance, that limit the fraction of administered NPs that reach the target therapeutic site.
- Immunogenicity: nano-DDSs must navigate the body’s immune system, which can recognize NPs as foreign objects and attempt to eliminate them. This immunogenicity can lead to premature clearance of the nanocarrier, reducing its efficacy before it reaches the target site. Overcoming this challenge often requires coating NPs with stealth materials (e.g., PEG), but the immune system can eventually recognize even these modified particles. Additionally, repeated exposure to certain NPs may induce an immune response, complicating long-term treatment.
- Long-term safety concerns: primarily due to their specific characteristics, NPs can have unexpected interactions with the body, including potential toxicity, immune responses, and accumulation in specific organs, even when designed for targeted delivery; this necessitates extensive research to fully understand the long-term effects of NPs in the body and to mitigate potential risks.
- Regulatory hurdles: The regulatory landscape for nano-DDSs is still evolving. Regulatory bodies like the US FDA and EMA require extensive safety, efficacy, and toxicity data to approve new DDSs, particularly when innovative nanomaterials are used. Nanoformulations must meet stringent criteria for clinical trials, often requiring additional studies on toxicity, biodistribution, and potential environmental impacts. Current regulatory frameworks may not be fully equipped to handle the complexities of nanomedicines, leading to delays in approval and market entry. The lack of standardized testing protocols for nanomaterials adds another layer of difficulty.
- Standardization issues: Lack of standardized methods for characterizing and manufacturing NPs lead to difficulties in comparing research findings, ensuring consistent quality across batches, and navigating regulatory approval processes due to variations in particle size, surface chemistry, and morphology across different production techniques.
- Complex manufacturing processes: Manufacturing challenges associated with nano-DDSs include difficulties in achieving consistent particle size and size distribution, controlling surface chemistry and morphology, ensuring sterility and purity, scaling up production to meet clinical needs, and developing reliable analytical methods to characterize the NPs, all while maintaining cost-effectiveness and minimizing potential toxicity concerns.
- Nano-DDSs require interdisciplinary collaboration because of the many different areas of expertise involved in their development: nanotechnology, materials science, pharmacology, engineering, biology, and chemistry.
- Cost considerations: Developing nano-DDSs can be expensive due to the specialized equipment, materials, and expertise required. Moreover, the clinical development process (including preclinical studies, clinical trials, and manufacturing) is costly and time-consuming. For widespread use, the cost of nano-DDSs needs to be justified by significant improvements in patient outcomes. High costs may limit accessibility, particularly in low-resource healthcare settings, and could deter investment from pharmaceutical companies if the market potential is uncertain.
- Inadequate understanding of pharmacokinetics: poor understanding of NP biodistribution, rapid clearance from the bloodstream, difficulty in achieving targeted delivery to specific tissues, inconsistent drug release profiles, potential for off-target accumulation, and variability in patient response due to differences in physiological conditions all can significantly impact the efficacy and safety of nanomedicines.
- Ethical concerns surrounding nano-DDSs primarily center around potential risks to human health, including potential toxicity from NPs, unequal access to these therapies, privacy concerns related to nanomaterial tracking, and the need for informed patient consent regarding the use of nanotechnology in their treatment, all while ensuring responsible research and development practices to mitigate these risks.
- Key market challenges and roadblocks related to nano-DDSs: high development costs, complex manufacturing processes, stringent regulatory hurdles, potential toxicity concerns, lack of standardized testing methods, difficulties in achieving targeted delivery, and the need for robust clinical trials to demonstrate efficacy and safety all can significantly impede the market adoption of nanomaterial-based drugs despite their potential therapeutic advantages.

Efforts are ongoing to overcome these challenges through continuous research, technological innovation, collaboration, and regulatory adaptation. As the field evolves, addressing these challenges will be crucial for realizing the full potential of nano-DDSs in improving drug efficacy and patient outcomes.

## Supporting Information Available

Landscape of the nano-DDSs research; Figure S1, early growth of the number of documents (journal articles and patents) related to nanosized DDS in the CAS Content Collection and Nano-DDS vs overall DDS-related documents yearly growth; Figure S2, Leading countries/regions with respect to the numbers of nano-DDS-related journal articles and patents; Figure S3, leading scientific journals with respect to the number of published nano-DDS-related articles and the average number of citations per article and classification of the top scientific journals with respect to the number of published nano-DDS-related articles and number of citations; Figure S4, leading academic research organizations with respect to the number of published nano-DDS-related articles and the number of citations per article; Figure S5, top academic and commercial organizations with respect to the number of nano-DDS-related patents; Figure S6, applications of the nano-DDS as reflected in the CAS Content Collection (PDF)

## Related Paper

Kevin J. Hughes, Magesh Ganesan, Rumiana Tenchov, Kavita A. Iyer, Krittika Ralhan, Leilani Lotti Diaz, Robert E. Bird, Julian Ivanov, Qiongqiong Zhou. Nanoscale materials at work: Mapping emerging applications in energy, medicine, and beyond. 2024. ChemRxiv. DOI: 10.26434/chemrxiv-2024-s75wv; https://chemrxiv.org/engage/chemrxiv/article-details/6622bd8891aefa6ce1ebc7f5 (accessed Dec 19, 2024).

## Vocabulary:

**Nanoparticle**: Particle with dimensions measured in nanometers (nm), typically ranging from 1 to 100 nm in size. Due to their small size, NPs exhibit distinctive physical and chemical properties that differ significantly from larger particles of the same material. These properties make them useful in various fields, including medicine, engineering, and environmental science.
**Nanocrystal**: A nanoscale crystal, with dimensions measured in nanometers (typically a few nanometers in size). Nanocrystals have a crystalline structure throughout their volume, with atoms arranged in a repeating, orderly pattern. This structure gives them specific optical, electrical, and magnetic properties, due to quantum confinement effects.
**Nanotube**: A nanoscale cylindrical structure with a hollow core. These structures can have diameters ranging from about 1 to tens of nanometers and lengths up to several millimeters. Carbon nanotubes (CNTs) are the most well-known type, consisting of rolled-up sheets of graphene. They exhibit remarkable properties, including high tensile strength, excellent thermal and electrical conductivity, and unique quantum effects.
**Quantum dot**: A nanoscale semiconductor particle, typically a few nanometers in size, that exhibits distinctive optical and electronic properties due to quantum mechanical effects. When illuminated by UV light, quantum dots can emit light of various colors depending on their size, a result of quantum confinement. These properties make quantum dots useful in applications such as medical imaging, display technologies, and quantum computing.
**Exosome**: A nanosized subset of extracellular vesicles (diameter ∼30–150 nm) comprising bioactive cargos, including proteins, nucleic acids, lipids, and metabolites.
