# Supplementary material for: Transforming Medicine: Cutting-Edge Applications of Nanoscale Materials in Drug Delivery
Source: ACS Nano. 2025 Jan 17;19(4):4011–38. doi: 10.1021/acsnano.4c09566 (PMC11803921; doi:10.1021/acsnano.4c09566)
Supplement: Supplementary file 1 — nn4c09566_si_001.pdf [file nn4c09566_si_001.pdf]

Supporting Information

Transforming medicine: cutting-edge applications of nanoscale materials in drug delivery

Rumiana Tenchov†[1], Kevin J. Hughes†[1], Magesh Ganesan†[2], Kavita A. Iyer†[1], Krittika Ralhan†[2], Leilani M Lotti Diaz†[1], Robert E. Bird†[1], Julian M. Ivanov[1], Qiongqiong Angela Zhou* [1]

[1]CAS, a division of the American Chemical Society, Columbus OH 43210, USA

[2]ACS International India Pvt. Ltd., Pune 411044, India

*Corresponding author: qzhou@cas.org

Landscape of the nano-sized drug delivery systems research

In recent years, sizeable methodological progress and a wealth of knowledge have promoted the advancement of research on nano-sized DDS, enhancing our understanding of their structure and efficiency. This is reflected in the consistent growth in the number of related scientific publications (journal articles and patents) in the last two decades (Figure S1A).

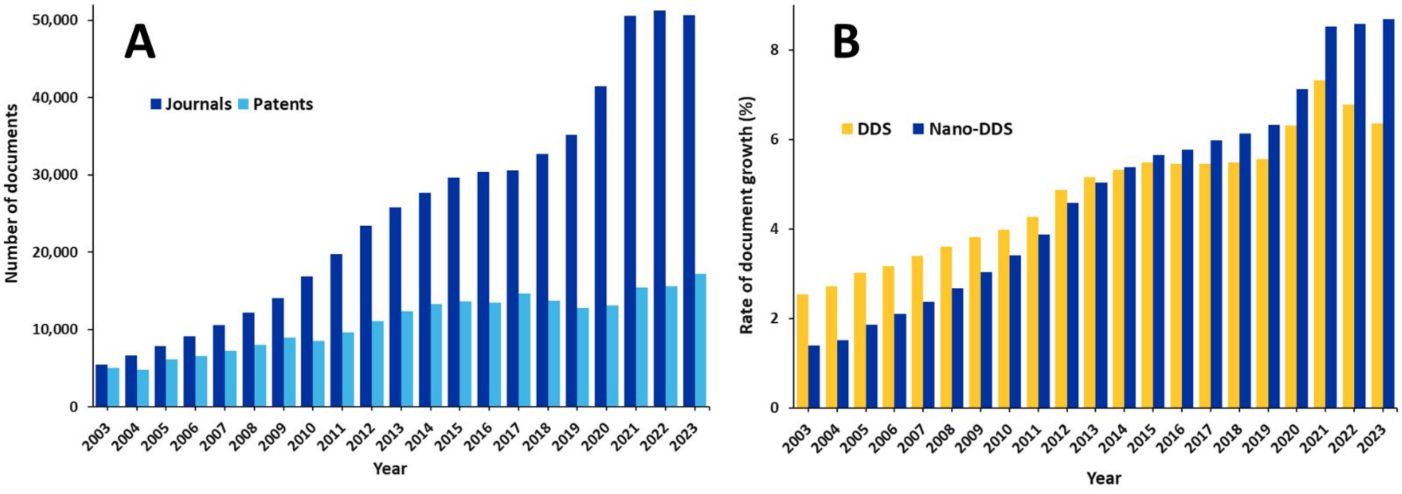

Figure S1. (A) Yearly growth of the number of documents (journal articles and patents) related to nanosized DDS in the CAS Content Collection; (B) Nano-DDS vs. overall DDS-related documents yearly growth.

In Figure S1B, the yearly growth rate of the number of publications in the CAS Content Collection related to nano-sized DDS are compared to those generally related to DDS. While in the years 2003-2013 the nano-DDS exhibit slower rate, during the last decade the number of publications related to nano-DDS has grown at similar or greater rates than the number of publications for DDS as a whole, with a notable increase in the last three years. The recognition of the potential advantages of nano-DDS over traditional DDS has likely driven increased interest in and publication rates for nano-DDS.

Currently, there are over 600,000 scientific publications (mainly journal articles and patents) in the CAS Content Collection related to nano-DDS. Journal article and patent publication counts have increased steadily over the last decades, with journal articles increasing by over 30% in the last three years (Figure S1A). Growth in the number of patents is slower when compared to journal publications and this is indicative of the field being in the phase of scientific knowledge accumulation often preceding subsequent transition into patentable and more commercial applications.

China, the United States, India, South Korea, and Japan, are the leaders with respect to the number of published journal articles and patents related to nano-DDS research, with China emerging as an eminent leader (Figure S2).

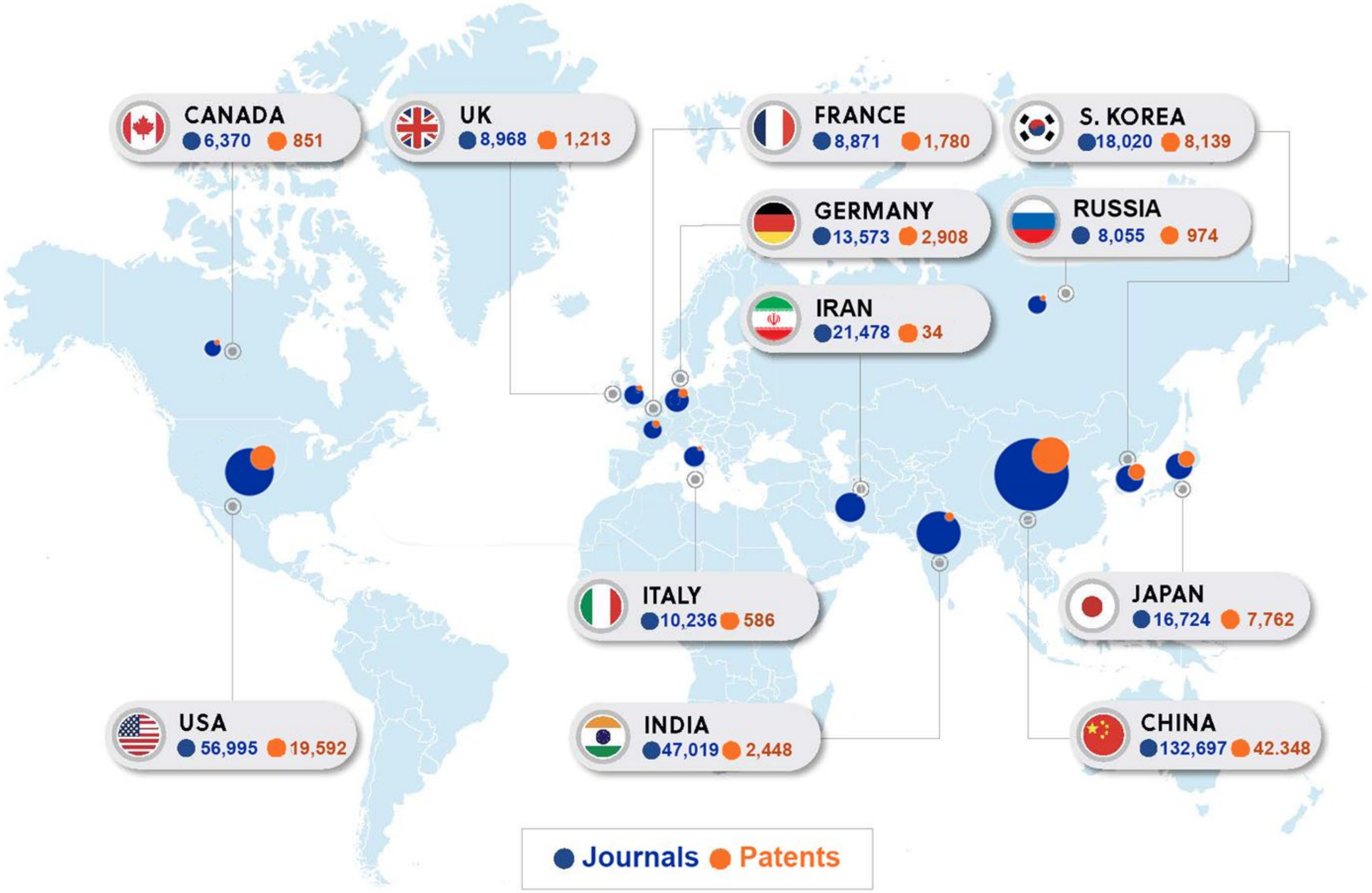

Figure S2. Leading countries/regions with respect to the numbers of nano-DDS-related journal articles (blue) and patents (orange). Data includes publications (journals and patents) from the CAS Content Collection for the period 2003-2023

The scientific journals *ACS Applied Materials & Interfaces, RSC Advances, Colloids & Surfaces, ACS Nano,* and *Nanoscale* have published the highest number of articles related to nano-DDS (Figure S3B), while *ACS Nano* and *Journal of the American Chemical Society (JACS)* lead in terms of number of citations (Figure S1C). When the citations per article, an indicator of the impact of journal publications, are considered, *JACS, Biomaterials*, and *ACS Nano* [1-11] emerge at the top (Figure S3A).

Ranking research institutions first by the volume of journal publications (Figure S2A) followed by the average number of citations per publication, considered as an indication of the scientific impact of a given publication (Figure S4B), allows the identification of leading academic organizations actively participating in the area of nano-DDS research. Three universities from the USA (Northwestern University, Stanford University, and the Massachusetts Institute of Technology) have the highest number of citations per publication (>100) indicating the high scientific impact of those publications (Figure S4B). Indeed, the International Institute of Nanotechnology [12] at Northwestern University (USA) is known for performing high impact nano-DDS-related research (see, e.g., [13-17]). One such highly cited article from that organization titled

“Surface Engineered Polymersomes for Enhanced Modulation of Dendritic Cells During Cardiovascular Immunotherapy” describes polymeric nanocarriers decorated with an optimized surface density of a lipid construct, to demonstrate the therapeutic augmentation and dosage lowering capability of cell-targeted nanotherapy in the treatment of cardiovascular disease. An article titled “PEGylated Nanographene Oxide for Delivery of Water-Insoluble Cancer Drugs” authored by researchers from Stanford University has received over 3,000 citations. [18] Another highly cited article: “Knocking down barriers: advances in siRNA delivery” authored by scientists at the Massachusetts Institute of Technology highlights novel synthetic materials for the encapsulation and intracellular delivery of siRNA. [19]

The University of California is the distinct leader with respect to the number of patents by academic organizations (Figure S5A). Among commercial organizations, F. Hoffmann-La Roche (Switzerland), Procter & Gamble (USA), and Novartis (Switzerland) are the companies with the highest number of nano-DDS-related patents (Figure S5B). Recent patents of F. Hoffmann-La Roche appear to be focused on cancer immunotherapy and hepatitis B therapy utilizing nucleic acid nanocarriers such as lipid vesicles[20] and hyaluronic acid conjugates,[21, 22] as well as highly porous polymer[23] and nanopore-based[24] diagnostic methods. Novartis, a Swiss pharmaceutical company, has filed patents in recent years that related to treatment of cancer, liver and infectious disease utilizing nanocarriers such as micelles, liposomes and emulsions,[25] mesoporous silica nanoparticles,[26] and nanostructured amorphous materials.[27] Patents of Procter & Gamble and L’Oreal relate mainly to nanodelivery in cosmetics, a burgeoning application for nano-DDS.

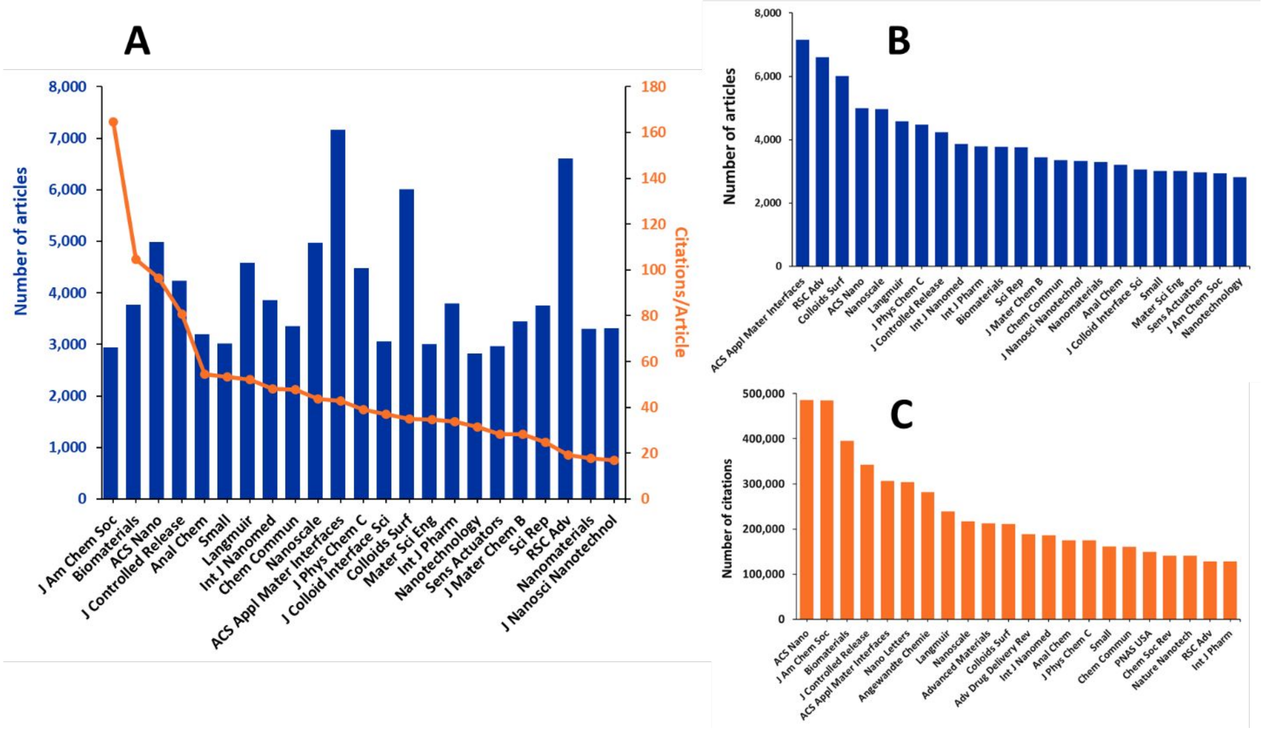

Figure S3. (A) Leading scientific journals with respect to the number of published nano-DDS-related articles (blue bars) and the average number of citations per article (orange line). This figure was made by first selecting the top 100 journals in terms of nano-DDS publications, then ranking them based on average citations per article. The top journals based on this ranking are

shown. Insets: Classification of the top scientific journals with respect to the number of published nano-DDS-related articles (B), and number of citations (C).

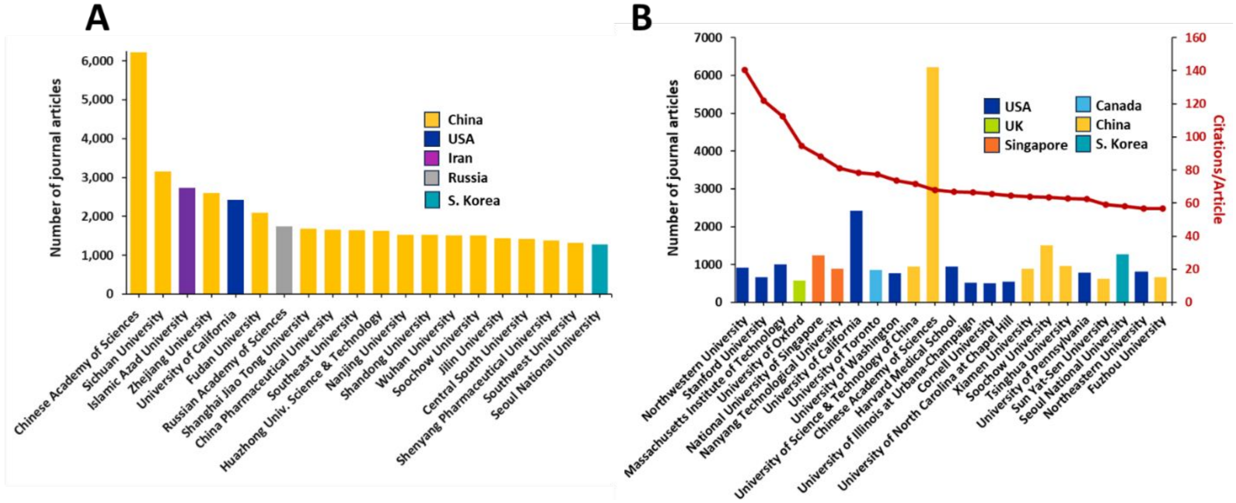

Figure S4. Leading academic research organizations with respect to the number of published nano-DDS-related articles (bars) (A), and the number of citations per article (red line) (B). The bars have been colored to represent different countries or regions as indicated by the legend. In (B), organizations were selected from the top 100 in terms of number of publications in the nano-DDS area.

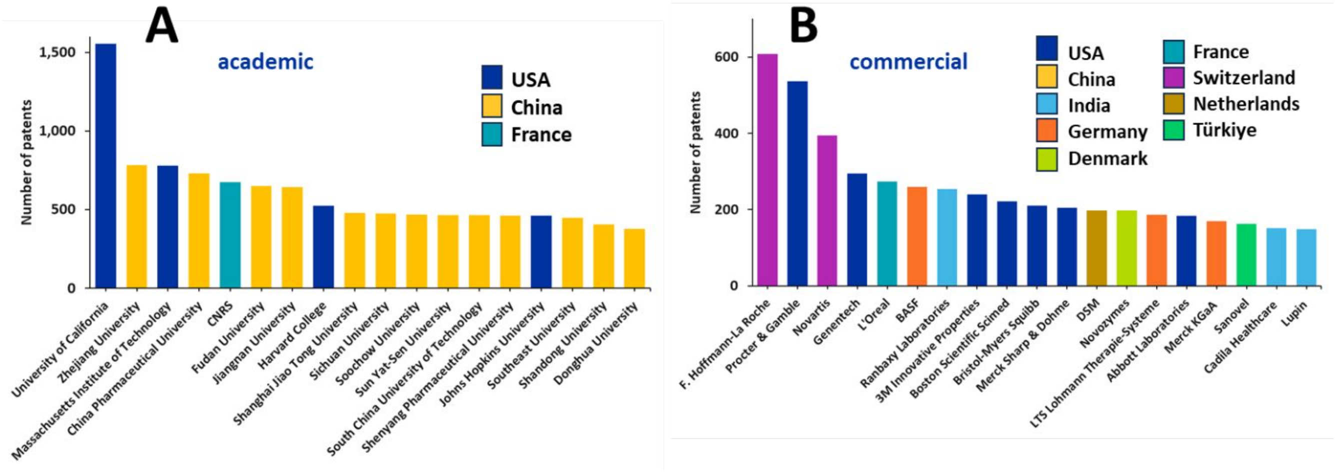

Figure S5. Top academic (A) and commercial (B) organizations with respect to the number of nano-DDS-related patents.

Figure S6A shows the percentage of documents – journal articles and patents – related to the various application fields, and Figure S6B presents the relative annual growth of those documents. As anticipated, the medical applications including drug/vaccine/gene delivery and diagnostic/imaging dominate, comprising in combination 91% of journal articles and 82% of patents.

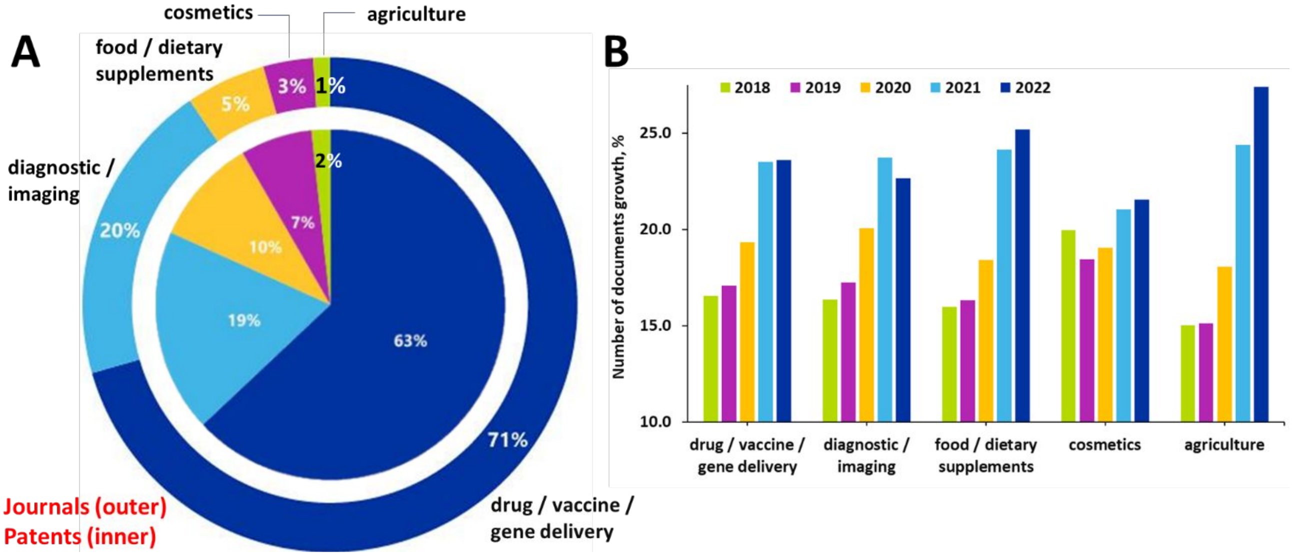

Figure S6. Applications of the nano-DDS as reflected in the CAS Content Collection: (A) Percentage of nano-DDS related documents: journals (outer circle) and patents (inner circle) in the period 2003-2022; (B) Yearly growth of the relative percentage of documents related to various nano-DDS applications for the 5-year period 2018-2022.

References:

1. Farokhzad, O. C., and Langer, R. (2009) Impact of Nanotechnology on Drug Delivery. ACS Nano 3, 16-20.
2. AshaRani, P. V., Low Kah Mun, G., Hande, M. P., and Valiyaveettil, S. (2009) Cytotoxicity and Genotoxicity of Silver Nanoparticles in Human Cells. ACS Nano 3, 279-290.
3. Liong, M., Lu, J., Kovochich, M., Xia, T., Ruehm, S. G., Nel, A. E., Tamanoi, F., and Zink, J. I. (2008) Multifunctional Inorganic Nanoparticles for Imaging, Targeting, and Drug Delivery. ACS Nano 2, 889-896.
4. Li, Y., Zhang, W., Niu, J., and Chen, Y. (2012) Mechanism of Photogenerated Reactive Oxygen Species and Correlation with the Antibacterial Properties of Engineered Metal-Oxide Nanoparticles. ACS Nano 6, 5164-5173.
5. Liu, Z., Sun, X., Nakayama-Ratchford, N., and Dai, H. (2007) Supramolecular Chemistry on Water-Soluble Carbon Nanotubes for Drug Loading and Delivery. ACS Nano 1, 50-56.
6. Tian, Q., Jiang, F., Zou, R., Liu, Q., Chen, Z., Zhu, M., Yang, S., Wang, J., Wang, J., and Hu, J. (2011) Hydrophilic Cu9S5 Nanocrystals: A Photothermal Agent with a 25.7% Heat Conversion Efficiency for Photothermal Ablation of Cancer Cells in Vivo. ACS Nano 5, 9761-9771.
7. Casals, E., Pfaller, T., Duschl, A., Oostingh, G. J., and Puntes, V. (2010) Time Evolution of the Nanoparticle Protein Corona. ACS Nano 4, 3623-3632.
8. Tian, B., Wang, C., Zhang, S., Feng, L., and Liu, Z. (2011) Photothermally Enhanced Photodynamic Therapy Delivered by Nano-Graphene Oxide. ACS Nano 5, 7000-7009.
9. Pelaz, B., Alexiou, C., Alvarez-Puebla, R. A., Alves, F., Andrews, A. M., Ashraf, S., Balogh, L. P., Ballerini, L., Bestetti, A., Brendel, C., et al. (2017) Diverse Applications of Nanomedicine. ACS Nano 11, 2313-2381.

10. Tenchov, R., Bird, R., Curtze, A. E., and Zhou, Q. (2021) Lipid Nanoparticles─From Liposomes to mRNA Vaccine Delivery, a Landscape of Research Diversity and Advancement. ACS Nano 15, 16982-17015.
11. Tenchov, R., Sasso, J. M., Wang, X., Liaw, W.-S., Chen, C.-A., and Zhou, Q. A. (2022) Exosomes─Nature's Lipid Nanoparticles, a Rising Star in Drug Delivery and Diagnostics. ACS Nano 16, 17802-17846.
12. International Institute of Nanotechnology https://www.iinano.org/ (accessed December 18, 2023).
13. Giljohann, D. A., Seferos, D. S., Daniel, W. L., Massich, M. D., Patel, P. C., and Mirkin, C. A. (2010) Gold Nanoparticles for Biology and Medicine. Angewandte Chemie International Edition 49, 3280-3294.
14. Yi, S., Zhang, X., Sangji, M. H., Liu, Y., Allen, S. D., Xiao, B., Bobbala, S., Braverman, C. L., Cai, L., Hecker, P. I., et al. (2019) Surface Engineered Polymersomes for Enhanced Modulation of Dendritic Cells During Cardiovascular Immunotherapy. Advanced Functional Materials 29, 1904399.
15. Park, S. Y., Lytton-Jean, A. K. R., Lee, B., Weigand, S., Schatz, G. C., and Mirkin, C. A. (2008) DNA-programmable nanoparticle crystallization. Nature 451, 553-556.
16. Marraffini, L. A., and Sontheimer, E. J. (2008) CRISPR Interference Limits Horizontal Gene Transfer in Staphylococci by Targeting DNA. Science (New York, N.Y.) 322, 1843-1845.
17. Cui, H., Webber, M. J., and Stupp, S. I. (2010) Self-assembly of peptide amphiphiles: From molecules to nanostructures to biomaterials. Pept. Sci. (Hoboken, NJ, U. S.) 94, 1-18.
18. Liu, Z., Robinson, J. T., Sun, X., and Dai, H. (2008) PEGylated Nanographene Oxide for Delivery of Water-Insoluble Cancer Drugs. Journal of the American Chemical Society 130, 10876-10877.
19. Whitehead, K. A., Langer, R., and Anderson, D. G. (2009) Knocking down barriers: advances in siRNA delivery. Nature reviews. Drug discovery 8, 129-138.
20. Grossen, P., and Keller, M. Lipid vesicle carrying nucleic acid as medicament, for oral drug delivery. WO2020152303, 2020.
21. Kelley, R. F., Jr., Mehta, S. C., Tesar, D. B., Hannoush, R., Hansen, S. T., Dengl, S., Kettenberger, H., and Huelsmann, P. M. Hyaluronic acid binding derivatives of versican (VG1) for long acting delivery of therapeutics. WO2022081835, 2022.
22. Dengl, S., Kelley, R. F., Kettenberger, H., Hannoush, R., Hansen, S. T., Huelsmann, P. M., Mehta, S. C., and Tesar, D. B. Non-covalent protein-hyaluronan conjugates for long-acting ocular delivery. WO2022079161, 2022.
23. Silvestre, M. E., and Hug, S. Method for preparing highly porous polymer particles for diagnostic applications. WO2018189287, 2018.
24. Crisalli, P., Gremyachinskiy, D., Heindl, D., Kuchelmeister, H., Schraeml, M., and Trans, A. Nanopore-based methods and compositions for assessing analyte-ligand interactions and analyte concentration in fluid solution. WO2019197590, 2019.
25. Bordawekar, M. S., Patidar, K., Patel, P., Shaikh Hamid, S. M., and Verma, D. D. Novel pharmaceutical formulations. WO2022195545, 2022.
26. Koshy, S. T., and Canham, S. M. Mesoporous silica particles compositions for viral delivery. WO2020176397, 2020.
27. Huang, D., Wieckhusen, D., and Miller, D. Amorphous nanostructured pharmaceutical materials. WO2018229626, 2018.
